# Microglia activation orchestrates CXCL10-mediated CD8^+^ T cell recruitment to promote aging-related white matter degeneration

**DOI:** 10.1038/s41593-025-01955-w

**Published:** 2025-05-22

**Authors:** Janos Groh, Ruoqing Feng, Xidi Yuan, Lu Liu, Dennis Klein, Gladis Hutahaean, Elisabeth Butz, Zhen Wang, Lisa Steinbrecher, Jonas Neher, Rudolf Martini, Mikael Simons

**Affiliations:** 1 Institute of Neuronal Cell Biology, Technical University Munich, Munich, Germany; 2 Department of Neurology, Section of Developmental Neurobiology, University Hospital Würzburg, Würzburg, Germany; 3 German Center for Neurodegenerative Diseases, Munich, Germany; 4 Munich Cluster of Systems Neurology, Munich, Germany; 5 Neuroimmunology and Neurodegenerative Disease, German Center for Neurodegenerative Diseases (DZNE), Tuebingen, Germany; 6 Department of Cellular Neurology, Hertie Institute for Clinical Brain Research, University of Tübingen, Tübingen, Germany

**Keywords:** Oligodendrocyte, Myelin biology and repair

## Abstract

Aging is the major risk factor for neurodegeneration and is associated with structural and functional alterations in white matter. Myelin is particularly vulnerable to aging, resulting in white matter-associated microglia activation. Here we used pharmacological and genetic approaches to investigate microglial functions related to aging-associated changes in myelinated axons of mice. Our results reveal that maladaptive microglia activation promotes the accumulation of harmful CD8^+^ T cells, leading to the degeneration of myelinated axons and subsequent impairment of brain function and behavior. We characterize glial heterogeneity and aging-related changes in white matter by single-cell and spatial transcriptomics and reveal elaborate glial–immune interactions. Mechanistically, we show that the CXCL10–CXCR3 axis is crucial for the recruitment and retention of CD8^+^ T cells in aged white matter, where they exert pathogenic effects. Our results indicate that myelin-related microglia dysfunction promotes adaptive immune reactions in aging and identify putative targets to mitigate their detrimental impact.

## Abstract (web summary)

White matter aging leads to degenerative and inflammatory changes. Here the authors show that microglial dysfunction exacerbates glial CXCL10 expression, harmful CD8^+^ T cell recruitment, myelinated axon damage and functional decline in aging mice.

## Main

Due to its limited capacity for regeneration, the brain must maintain its functionality throughout an entire lifetime. However, with aging, the functional capabilities of the brain inevitably change, leading to a decline in cognitive abilities^1,2^. One structure especially susceptible to aging-related changes is the white matter, which is mainly composed of myelinated axons. This vulnerability might stem from the unique anatomical features of oligodendrocytes, which have an extensively enlarged surface area with numerous branching processes that create multiple layers of myelin sheaths around axons^3^. Within the myelin sheaths, the membrane components are enclosed in densely packed layers that experience minimal turnover, making them more prone to accumulating oxidative damage associated with aging^4^.

As we age, white matter decreases in volume, loses its microstructural properties and accumulates focal lesions^5,6^. On magnetic resonance imaging, these lesions appear as white matter hyperintensities, which mainly reflect small vessel pathology, myelin alterations and axonal loss. Additionally, diffusion tensor imaging shows impairments in white matter tract integrity. Ultrastructural analyses reveal the pathology of myelinated fibers, including focal areas of decompacted, redundant and degenerated myelin, along with axonal damage^7,8^. The structural, molecular and cellular changes associated with aging-related white matter degeneration are complex and result from a combination of various factors^8^. Understanding these processes is crucial as they contribute to a decline in cognitive functions and serve as a risk factor for neurodegenerative diseases, increasing susceptibility to stroke and dementia^9^. White matter changes and oligodendrocyte dysfunction in aging lead to neuroinflammation, particularly through the activation of microglia, which are responsible for clearing aberrant myelin^10–14^. This function of microglia is crucial for maintaining and protecting the structural and functional integrity of the myelin sheath. However, progressive age-related myelin breakdown can overwhelm the phagocytic function of microglia^12,15^.

Age-related changes in myelin integrity lead to a distinct state of microglia in white matter^16^. Specifically, these white matter-associated microglia (WAM) display characteristics akin to disease-associated microglia (DAM) or the microglia-neurodegenerative phenotype, demonstrating partial activation of the transcriptional program associated with disease^17,18^. Some of these cells form nodules, clusters of several closely associated cells that are actively involved in clearing aberrant myelin^12,16^. Additionally, there are microglia characterized by the accumulation of lipid droplets, impaired phagocytosis, high levels of reactive oxygen species and secretion of pro-inflammatory cytokines in the aging brain^19,20^. Others display signs of microglial dysfunction, such as the accumulation of insoluble lysosomal inclusions resembling lipofuscin, as well as dystrophic changes like cytoplasmic spheroids, beading and fragmentation^12,21^. These findings underscore various forms of microglial reactivity and raise questions about their roles in white matter pathology affecting myelinated axons. Do microglia primarily function adaptively and protectively against aging-related myelin damage? Or are there also maladaptive responses that contribute to white matter pathology? What is the relationship between aging-related microglial reactivity and the accumulation of CD8^+^ T cells, known to drive axon degeneration and myelinating oligodendrocyte loss?^22,23^

Here we used pharmacological and genetic tools to study microglial functions in aging-related changes of myelinated axons in the white matter in mice. Our findings demonstrate that the maladaptive activation of microglia facilitates the accumulation of CD8^+^ T cells, resulting in damage to myelinated axons and the impairment of brain function. Using single-cell and spatial transcriptomics multiplexed error-robust fluorescence in situ hybridization (MERFISH), we characterize the diversity of glial cells and age-related changes in white matter, revealing complex interactions between glial cells and the immune system. We identify the CXCL10–CXCR3 axis as critical in recruiting and retaining CD8^+^ T cells in aged white matter, where they exert pathogenic effects. These findings suggest that dysfunctional microglia related to myelin changes promote adaptive immune responses during aging, highlighting potential targets for mitigating their harmful effects.

## Results

### Maladaptive microglia activation worsens white matter aging

**Extended Data Fig. 1:**
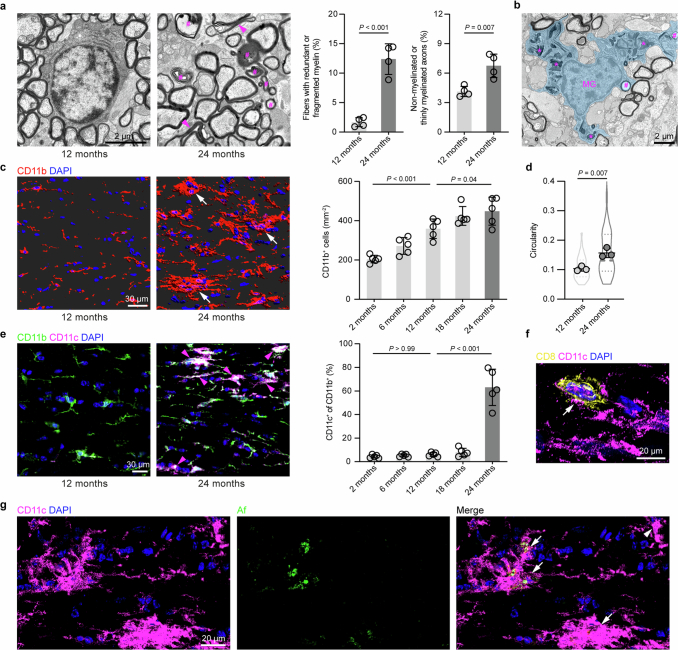
Myelin alterations and microglial activation in aged optic nerves. **a**, Representative electron micrographs and quantification of myelin alterations in optic nerve cross-sections from adult (12-month-old) and aged (24-month-old) mice. Scale bar, 2 μm. Fibers ensheathed with redundant myelin (arrowheads) or myelin fragments (hashtags), as well as nonmyelinated or thinly myelinated axons, accumulate in aged nerves (each circle represents the mean value of one mouse; *n* = 4 mice per group, unpaired two-sided Student’s *t*-test: *t* = 7.916, df = 6). **b**, Representative electron micrograph of a microglial cell (MG; light blue pseudocolor) in an optic nerve cross-section from an aged mouse demonstrates intracellular accumulation of myelin fragments (hashtags) and lysosomal storage material (asterisks). Scale bar, 2 μm. **c**, Immunofluorescence detection, IMARIS Z-stack surface rendering and quantification of CD11b^+^ microglia in optic nerves of mice at various ages (*n* = 5 mice per group, one-way ANOVA with Bonferroni’s multiple comparisons test, *F*(4, 20) = 21.92, *P* < 0.001). Arrows indicate groups of several microglia clustered in nodules. Scale bar, 30 µm. **d**, Microglial form factor analysis demonstrates increased circularity in optic nerves from aged mice (*n* = 3 mice per group, unpaired two-sided Student’s *t*-test: *t* = 5.069, df = 4). **e**, Immunofluorescence detection and quantification of CD11c reactivity on CD11b^+^ microglia in optic nerves of adult and aged mice (*n* = 4 mice per group, one-way ANOVA with Bonferroni’s multiple comparisons test, *F*(4, 20) = 21.92, *P* < 0.001). Arrows indicate groups of several microglia clustered in nodules. Scale bar, 30 µm. **f**, Representative immunofluorescence detection of a CD8^+^ T cell in contact (arrow) with a CD11c^+^ microglial cell in the optic nerve of an aged mouse. Scale bar, 20 µm. **g**, Immunofluorescence detection of CD11c^+^-activated microglia and autofluorescent storage material (Af) in the optic nerve of an aged mouse. Microglia clustered in nodules often express CD11c and contain autofluorescent storage material (arrows), while single CD11c^+^ microglia usually display less autofluorescent storage material (arrowheads). Scale bar, 20 µm. Data are presented as the mean ± s.d. Source data

**Extended Data Fig. 2:**
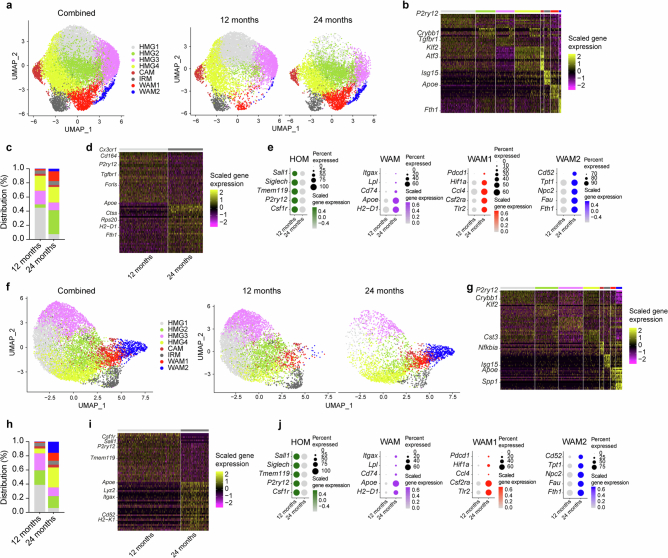
scRNA-seq reveals aging-related microglial signatures. **a**, UMAP visualization of live microglia sorted from adult (10-month-old) and aged (24-month-old) mouse brains and analyzed by scRNA-seq. Combined (left, 15,843 cells) and separate (right) visualizations of cells from adult (9,203 cells) and aged (6,640 cells) brains are displayed (*n* = 3 mice per group). **b**, Heatmap of top 10 cluster-specific genes. The color scale is based on a z-score distribution from −2 (purple) to 2 (yellow). **c**, Distribution of different cluster frequencies among all microglia. **d**, Heatmap of top 30 differentially expressed genes comparing microglia isolated from adult and aged brains across all clusters as identified in **a**. **e**, Dot plot expression visualization of selected genes implicated in microglial homeostasis (HOM) and activation (WAM), as well as selected marker genes of the distinct WAM states across all microglia clusters. The color scales are based on z-score distributions. **f**, UMAP visualization of fixed microglia sorted from adult (12-month-old) and aged (24-month-old) mouse brains and analyzed by scRNA-seq. Combined (left, 9,925 cells) and separate (right) visualizations of cells from adult (6,858 cells) and aged (3,067 cells) brains are displayed (*n* = 2 mice per group). **g**, Heatmap of top 10 cluster-specific genes. The color scale is based on a z-score distribution from −2 (purple) to 2 (yellow). **h**, Distribution of different cluster frequencies among all microglia. **i**, Heatmap of top 30 differentially expressed genes comparing microglia isolated from adult and aged brains across all clusters as identified in **a**. **j**, Dot plot expression visualization of selected genes implicated in microglial homeostasis (HOM) and activation (WAM), as well as selected marker genes of the distinct WAM states across all microglia clusters. The color scales are based on z-score distributions. HMG, homeostatic microglia; CAM, capillary-associated microglia; IRM, interferon-responsive microglia; WAM, white matter-associated microglia.

**Extended Data Fig. 3:**
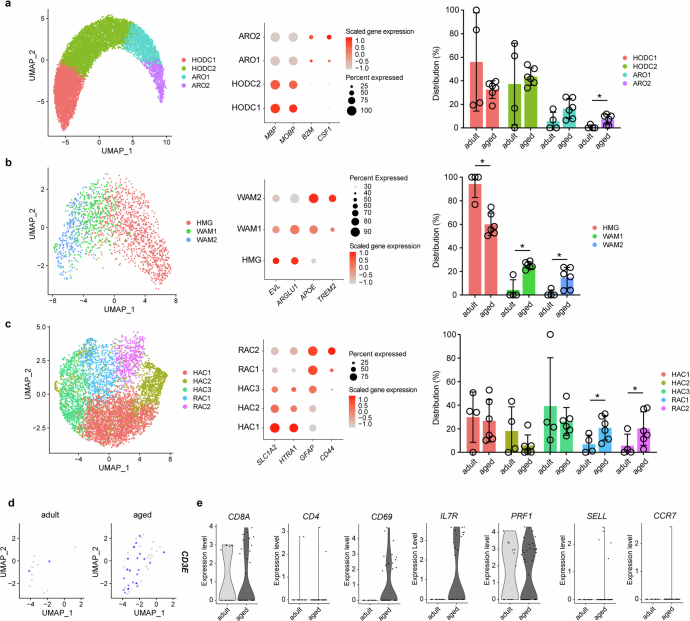
scRNA-seq reveals aging-related glial and T cell signatures in human brain tissue. **a**–**c**, UMAP visualization (left), dot plot expression of selected marker genes (middle) and distribution (right) of fixed oligodendrocytes (11,221 cells; **a**), microglia (1,768 cells; **b**) and astrocytes (6,354 cells; **c**) from adult (25–50-year-old, *n* = 4 samples) and aged ( > 70-year-old, *n* = 6 samples) human brain tissue (BA24) analyzed by scRNA-seq. Asterisks indicate significant differences identified by scCODA. **d**, UMAP visualizations of *CD3E* gene expression for a T cell cluster identified in adult (left) and aged (right) human brain tissue. **e**, Violin plots showing the expression of selected genes indicating T cell subtype, tissue residency, memory, effector or egress function in T cells from adult and aged human brain tissue.

To characterize aging-related changes in myelin integrity, we focused on the mouse optic nerve, a well-characterized, accessible and compartmentalized white matter tract of the central nervous system (CNS). As shown previously^22^, we detected myelin perturbation with increased frequencies of fibers ensheathed with redundant or fragmented myelin as well as mild demyelination in aged (24-month-old) compared with adult (12-month-old) mice (Extended Data Fig. 1a). Furthermore, using electron microscopy and immunofluorescence, we observed morphological changes of microglia in aged optic nerves, which included the presence of myelin fragments or lipofuscin-like lysosomal storage material (Extended Data Fig. 1b–g). Similar to the corpus callosum^12,16^, microglia displayed activation characterized by reduced branching and increased circularity, clustering in nodules (Supplementary Video) and a steep rise in CD11c expression between 18 and 24 months of age (Extended Data Fig. 1c–e). CD8^+^ T cells, typically accumulating in aged white matter^22,23^, were frequently (~40%) contacting CD11c^+^ activated microglia, irrespective of their arrangement in single cells or clusters. Thus, we confirm and extend previous knowledge of white matter aging and show that its features are also evident in the optic nerve. Using single-cell RNA sequencing (scRNA-seq) transcriptional analysis, we detected several clusters of homeostatic microglia and two distinct clusters of aging-enriched WAM that exhibited transcriptional profiles resembling those of DAM as reported previously^16^ (Extended Data Fig. 2a–e). Both WAM clusters were also detected when we applied a protocol for the isolation and enrichment of microglia and oligodendrocytes from paraformaldehyde (PFA)-fixed mouse brains, thereby excluding ex vivo dissociation artifacts^24^ (Extended Data Fig. 2f–j and Supplementary Figs. 1 and 2). Flow cytometry, immunofluorescence and electron microscopy supported the observation of distinct WAM states (Extended Data Fig. 3).

To confirm that aging-related glial activation and an accumulation of T cells also occur in humans, we performed fixed scRNA-seq of frozen brain autopsy samples from adult (25–50-year-old) and older (>70 years) humans without neurological disease^22^. We isolated fixed cells from the anterior cingulate cortex (BA24), a strongly myelinated region important for cognition and motor organization directly adjacent to the corpus callosum. After quality control and filtering, we subclustered oligodendrocytes, microglia and astrocytes from adult and aged samples based on marker gene expression (Supplementary Fig. 3a–c). Consistent with our observations in mice, we detected aging-related pro-inflammatory reactions in all glial cells and an accumulation of mostly CD8^+^ T cells with a tissue-resident memory (TRM) profile in human brain samples (Extended Data Fig. 3).

**Fig. 1:**
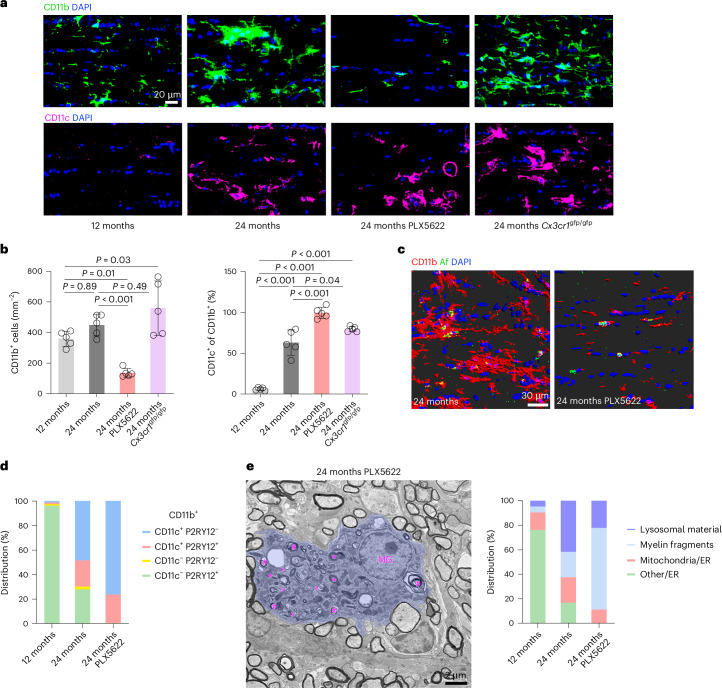
Microglial response to PLX5622 treatment or CX3CR1 deficiency in aged optic nerves. **a**,**b**, Immunofluorescence detection (**a**) and quantification (**b**) of CD11b^+^ (top) or CD11c^+^ (bottom) microglia (percentage of CD11b^+^) in longitudinal optic nerve sections from adult (12-month-old), aged (24-month-old), aged PLX5622-treated and aged *Cx3cr1*^gfp/gfp^ mice (each circle represents the mean value of one mouse; *n* = 5 mice per group, one-way ANOVA with Bonferroni’s multiple comparisons test, top—*F*(3, 16) = 16.15, *P* < 0.001; bottom—*F*(3, 16) = 108.8, *P* < 0.001). Scale bar, 20 μm. **c**, Immunofluorescence detection and IMARIS Z-stack surface rendering of CD11b^+^ microglia and autofluorescent storage material (Af) in optic nerves of aged and PLX5622-treated aged mice. Microglia resisting PLX5622 depletion frequently contain autofluorescent storage material. Scale bar, 30 µm. **d**, Distribution of CD11c/P2RY12 reactivity on microglia (percentage of CD11b^+^) in the optic nerves of adult, aged and aged PLX5622-treated mice as shown in Extended Data Fig. 3b (*n* = 5 mice per group). **e**, Representative electron micrograph (left) of a MG cell (blue pseudocolor) in an optic nerve cross-section from an aged PLX5622-treated mouse demonstrates ameboid morphology and intracellular accumulation of myelin fragments (hashtags), lysosomal storage material (asterisks) and few small lipid droplets (open arrowheads). Scale bar, 2 μm. Distribution (right) of MG grouped according to distinct ultrastructural characteristics as shown in Extended Data Fig. 3c (*n* = 4 mice per group). PLX5622 treatment in aged mice leads to a predominance of remaining microglia with myelin fragments and lysosomal storage material. Data are presented as the mean ± s.d. MG, microglia. Source data

**Fig. 2:**
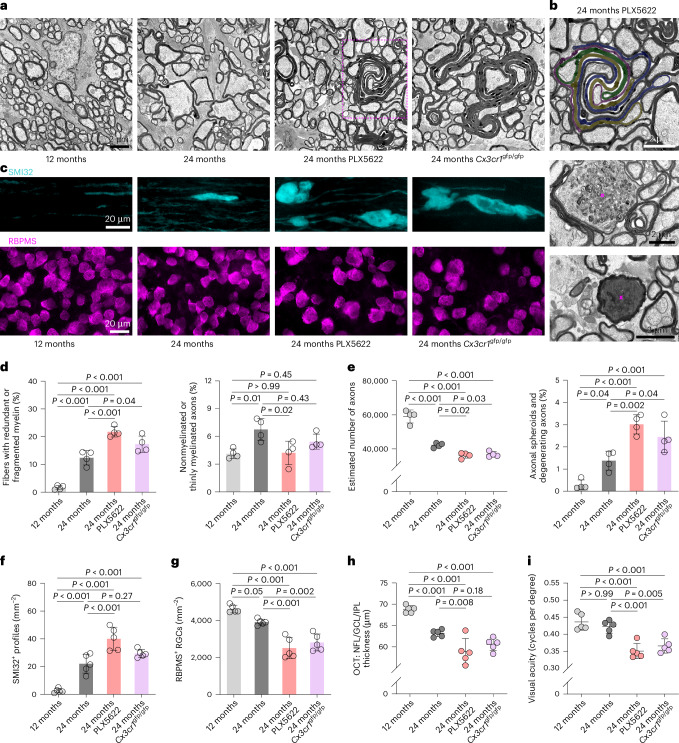
PLX5622 treatment or CX3CR1 deficiency aggravates aging-related myelin perturbation and axon degeneration. **a**, Representative electron micrographs of optic nerve cross-sections from adult (12-month-old), aged (24-month-old), aged PLX5622-treated and aged *Cx3cr1*^gfp/gfp^ mice. Scale bar, 2 μm. **b**, Electron micrographs of optic nerves from aged PLX5622-treated mice. Top, several axons are ensheathed with intertwined redundant myelin figures (individual redundant sheaths are pseudocolored). Middle, a myelinated axon showing accumulation of organelles and spheroid formation (circle). Bottom, a myelinated axon with dark cytoplasm undergoing degeneration (x). Scale bars, 2 µm. **c**, Immunofluorescence detection of SMI32^+^ axonal spheroids in longitudinal optic nerve sections (top) and RBPMS^+^ retinal ganglion cells in flat mount preparations (bottom) from adult, aged, aged PLX5622-treated and aged *Cx3cr1*^gfp/gfp^ mice. Scale bars, 20 μm. **d**, Electron microscopy-based quantification of fibers ensheathed with redundant or fragmented myelin (left) or thinly myelinated (*g* ratio ≥ 0.85) and nonmyelinated axons (right; each circle represents the mean value of one mouse; *n* = 4 mice per group, *F*(3, 12) = 61.25, *P* < 0.001). **e**, Electron microscopy-based estimation of total axonal numbers in the optic nerves (left) and quantification of fibers showing axonal spheroid formation or axon degeneration (right; *n* = 4 mice per group, *F*(3, 12) = 25.13, *P* < 0.001). **f**,**g**, Quantification of SMI32^+^ axonal spheroids (**f**) and RBPMS^+^ retinal ganglion cells (**g**; *n* = 5 mice per group, left—*F*(3, 16) = 39.95, *P* < 0.001; right—*F*(3, 16) = 32.14, *P* < 0.001). **h**, OCT analysis of the innermost retinal composite layer (NFL/GCL/IPL) thickness in peripapillary circle scans (*n* = 5 mice per group, *F*(3, 16) = 28.85, *P* < 0.001). **i**, Automated optokinetic response analysis of visual acuity (cycles per degree) shows a decline of visual acuity in PLX5622-treated or CX3CR1-deficient aged mice (*F*(3, 16) = 18.24, *P* < 0.001). All statistical comparisons were performed with one-way ANOVA with Bonferroni’s multiple comparison tests. Data are presented as the mean ± s.d. Source data

**Extended Data Fig. 4:**
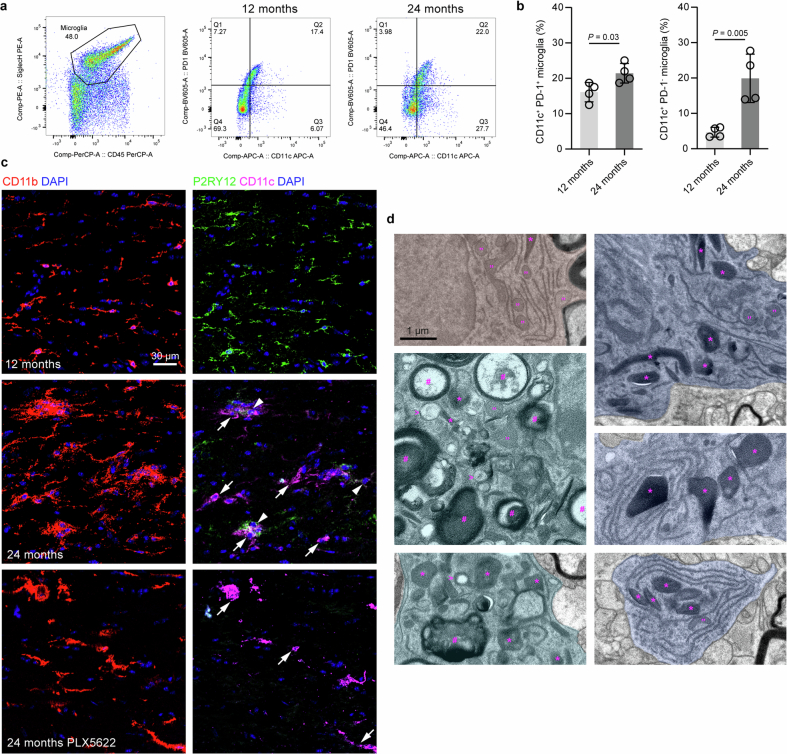
Validation of distinct microglial states in aged and PLX5622-treated aged brains. **a**, Representative flow cytometry plots for gating and analysis of single, viable CD45^low^Siglec-H^+^ microglia from adult (12-month-old) and aged (24-month-old) brains. WAM subsets comprise CD11c^+^PD-1^+^ (WAM1) and CD11c^+^PD-1^−^ (WAM2) cells. **b**, Flow cytometry-based quantification of CD11c^+^PD-1^+^ (WAM1, left) and CD11c^+^PD-1^−^ (WAM2, right) populations among microglia (each circle represents the mean value of one mouse; *n* = 4 mice per group, unpaired two-sided Student’s *t*-test, left: *t* = 2.735, df = 6, right: *t* = 4.358, df = 6). **c**, Representative immunofluorescence detection of CD11b in combination with CD11c and P2RY12 in optic nerves from adult, aged and PLX5622-treated aged mice. The arrowhead indicates a CD11b^+^CD11c^+^P2RY12^+^ (WAM1) cell and the arrows indicate CD11b^+^CD11c^+^P2RY12^−^ (WAM2) cells. Scale bar, 30 µm. **d**, Representative electron micrographs of microglia (pseudocolored according to different categories) in optic nerves from aged and PLX5622-treated aged mice demonstrate distinct ultrastructural characteristics comprising high mitochondria (quotation marks) and endoplasmic reticulum (ER) content, intracellular accumulation of myelin fragments (hashtags), few small lipid droplets (open arrowheads) and lysosomal storage material (asterisks). Scale bar, 1 µm. Data are presented as the mean ± s.d. Source data

**Extended Data Fig. 5:**
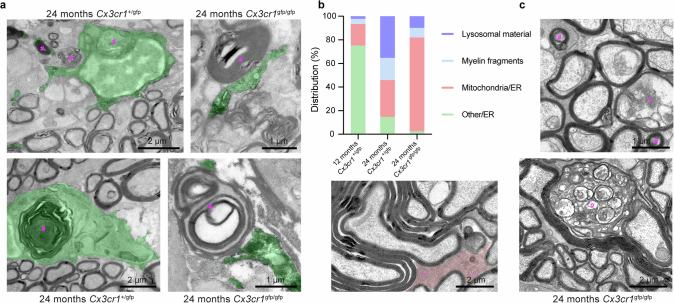
Dysfunctional uptake of perturbed myelin by CX3CR1-deficient microglia in aged white matter. **a**, Representative electron micrographs of microglial cells (green pseudocolor) in optic nerves from aged (24-month-old) *Cx3cr1*^+/gfp^ (left) and *Cx3cr1*^gfp/gfp^ (right) mice identified by GFP-directed immunolabeling (dark precipitate). Myelin fragments (hashtags) are often contacted but not ingested by CX3CR1-deficient microglia. Scale bars, 2 µm (left) and 1 µm (right). **b**, Distribution (top) of microglia in optic nerves from adult and aged *Cx3cr1*^+/gfp^ and aged *Cx3cr1*^gfp/gfp^ mice grouped according to distinct ultrastructural characteristics as shown in Extended Data Fig. 4d (*n* = 3 mice per group). Representative electron micrograph of a mitochondria-rich (quotation marks) microglial cell (red pseudocolor) in the optic nerve from an aged *Cx3cr1*^gfp/gfp^ mouse in contact with redundant myelin figures. **c**, Electron micrographs of optic nerves from aged *Cx3cr1*^gfp/gfp^ mice: myelin fragments (hashtags), myelinated axons showing signs of degeneration (x) and accumulation of organelles and spheroid formation (circle) are detectable. Scale bars, 1 µm (top) and 2 µm (bottom). Source data

To examine the role of microglia in white matter aging, we used the small molecule inhibitor PLX5622 to chronically ablate CSF-1R-dependent myeloid cells from the CNS^25^. Additionally, we studied aged CX3CR1-deficient (*Cx3cr1*^gfp/gfp^) mice. The rationale for using CX3CR1-deficient mice is based on the crucial role of the chemokine fractalkine (CX3CL1) and its receptor, CX3CR1, in neuroinflammation associated with neurodegenerative and demyelinating conditions^26,27^. Continuous administration of PLX5622 from 18 to 24 months of age resulted in a partial depletion of microglia, while CX3CR1 deficiency did not affect microglia number (Fig. 1a,b). Consistent with the increased resistance of activated microglia to pharmacological depletion, virtually all the remaining microglia in optic nerves from PLX5622-treated aged mice showed increased CD11c expression and less ramified morphology (Fig. 1b and Supplementary Fig. 4a,b). Moreover, the remaining microglia often contained autofluorescent lysosomal storage material (Fig. 1c) and lacked expression of the homeostatic marker P2RY12 (Fig. 1d and Extended Data Fig. 4b). Electron microscopy confirmed excessive amounts of myelin fragments and lysosomal storage material in the remaining microglia after PLX5622 treatment (Fig. 1e and Extended Data Fig. 4c). In contrast, microglia in nerves of aged *Cx3cr1*^gfp/gfp^ mice also showed elevated CD11c expression while retaining a more ramified morphology (Fig. 1a,b and Supplementary Fig. 4a,b). Using green fluorescent protein (GFP)-directed immunoelectron microscopy, we found that CX3CR1-deficient microglia often contacted redundant or fragmented myelin but failed to ingest it (Extended Data Fig. 5). Detailed morphometric quantification using electron microscopy showed a substantial increase in the frequency of fibers ensheathed with redundant or fragmented myelin following PLX5622 treatment or CX3CR1 deficiency in aged optic nerves (Fig. 2a,b,d). This resulted in the formation of elaborate, intertwined figures of redundant myelin. Additionally, PLX5622 treatment prevented the slight rise in nonmyelinated but not thinly myelinated axons observed in aged mice (Fig. 2d and Supplementary Fig. 4c). Strikingly, we also detected a substantially increased loss of axons and more ongoing axonal spheroid formation and degeneration (Fig. 2e). Corroborating our electron microscopic observations, axonal spheroids marked by nonphosphorylated neurofilaments (SMI32^+^) were significantly enriched in optic nerves from aged PLX5622-treated or *Cx3cr1*^gfp/gfp^ mice (Fig. 2c,f). Enhanced axon degeneration resulted in a stronger loss of RBPMS^+^ retinal ganglion cells (RGCs; Fig. 2g) and increased thinning of the inner retinal layers, as measured with optical coherence tomography (OCT; Fig. 2h). Behaviorally, PLX5622 treatment or CX3CR1 deficiency in aged mice resulted in decreased visual acuity, a deficit normally observed at even more advanced age^22^ (Fig. 2i).

**Fig. 3:**
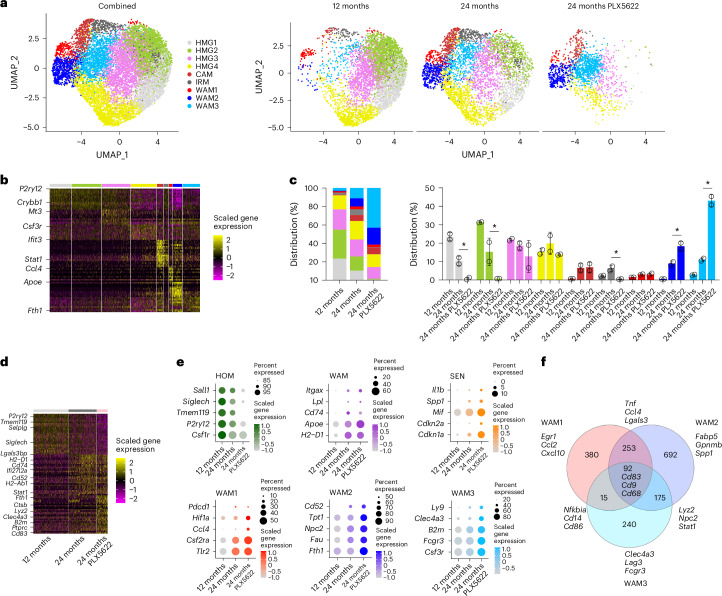
scRNA-seq reveals aging-related microglial signatures upon PLX5622 treatment. **a**, UMAP visualization of fixed microglia sorted from adult (12-month-old), aged (24-month-old) and PLX5622-treated aged mouse brains and analyzed by scRNA-seq. Combined (left, 11,201 cells) and separate (right) visualizations of cells from adult (5,293 cells), aged (4,312 cells) and PLX5622-treated aged (1,596 cells) brains are displayed. **b**, Heatmap of top ten cluster-specific genes. The color scale is based on a *z-*score distribution from −2 (purple) to 2 (yellow). **c**, Distribution of different cluster frequencies among all microglia and compositional comparison between samples (each circle represents one reaction; *n* = 2 reactions per group with two mice per reaction). WAMs are resistant to depletion and predominate after PLX5622 treatment in aged mice. Asterisks indicate significant differences identified by scCODA. Data are presented as the mean ± s.d. **d**, Heatmap of top 30 differentially expressed genes comparing microglia isolated from adult, aged and PLX5622-treated aged brains across all clusters as identified in **a**. **e**, Dot plot expression visualization of selected genes implicated in microglial homeostasis (HOM), activation (WAM) and senescence (SEN), as well as selected marker genes of the distinct WAM states across all microglia clusters. The color scales are based on *z-*score distributions. **f**, Venn diagram showing the overlap between marker genes of the distinct WAM states; selected genes are indicated. Complete lists of cluster-enriched markers and differentially expressed genes can be found in Supplementary Table 2. HMG, homeostatic microglia; CAM, capillary-associated microglia; IRM, IFN-responsive microglia.

To further characterize glial responses upon PLX5622 treatment in aging, we isolated and sorted single non-neuronal (NeuN^−^ DAPI^+^) cells from fixed mouse brain tissue for scRNA-seq (Supplementary Fig. 5). After quality control and filtering, we subclustered microglia from adult, aged and PLX5622-treated aged mice (Fig. 3a). The inclusion of the microglia depletion group resulted in the formation of an additional, third WAM cluster, which seemed to represent a hybrid state, showing differentially expressed genes characteristic of both previously identified WAM subsets and substantially accumulated in aged mice after PLX5622 treatment (Fig. 3a–c). One WAM cluster showed enrichment of pathways related to inflammatory response, leukocyte activation, lipoprotein clearance, glycolysis and NF-κB signaling. The other WAM cluster displayed enrichment of pathways related to ribosome assembly, oxidative phosphorylation, phagocytosis, response to type 2 interferon (IFN) and iron uptake. The third, PLX5622-enriched cluster showed pathways related to inflammatory response, antigen processing and presentation, T cell-mediated immunity and oxidative phosphorylation (Supplementary Data 1 and 2). Corroborating our immunohistochemical findings, inhibiting CSF-1R led to a preferential reduction in homeostatic microglia and increased expression of genes associated with activation and senescence (Fig. 3d–f). The resistance of the distinct microglia clusters to CSF-1R inhibition corresponded with their downregulation of *Csf1r* and the increased expression of genes encoding alternative receptors that support their survival (Supplementary Fig. 5d,e). Additionally, the remaining microglia showed upregulated expression of genes related to pro-inflammatory activation, T cell stimulation, lysosomal dysfunction and iron uptake (Fig. 3d–f).

**Extended Data Fig. 6:**
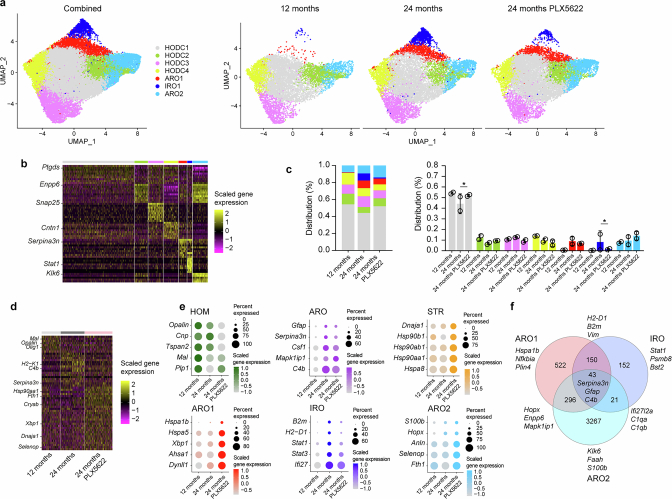
scRNA-seq reveals aging-related oligodendrocyte signatures upon PLX5622 treatment. **a**, UMAP visualization of fixed oligodendrocytes sorted from adult (12-month-old), aged (24-month-old) and PLX5622-treated aged mouse brains and analyzed by scRNA-seq. Combined (left, 30,110 cells) and separate (right) visualizations of cells from adult (8,052 cells), aged (10,173 cells) and PLX5622-treated aged (11,885 cells) brains are displayed. **b**, Heatmap of top 10 cluster-specific genes. The color scale is based on a z-score distribution from −2 (purple) to 2 (yellow). **c**, Distribution of different cluster frequencies among all oligodendrocytes and compositional comparison between samples (each circle represents one reaction; *n* = 2 reactions per group with 2 mice per reaction). Asterisks indicate significant differences identified by scCODA. Data are presented as the mean ± s.d. **d**, Heatmap of top 30 differentially expressed genes comparing oligodendrocytes isolated from adult, aged and PLX5622-treated aged brains across all clusters as identified in **a**. **e**, Dot plot expression visualization of selected genes implicated in oligodendrocyte homeostasis (HOM), aging response (ARO) and cell stress (STR), as well as selected marker genes of the distinct ARO and interferon-stimulated (IRO) states across all oligodendrocyte clusters. The color scales are based on z-score distributions. **f**, Venn diagram showing the overlap between marker genes of the distinct ARO and IRO states; selected genes are indicated. HODC, homeostatic oligodendrocytes; ARO, aging-related oligodendrocytes; IRO, interferon-responsive oligodendrocytes.

**Extended Data Fig. 7:**
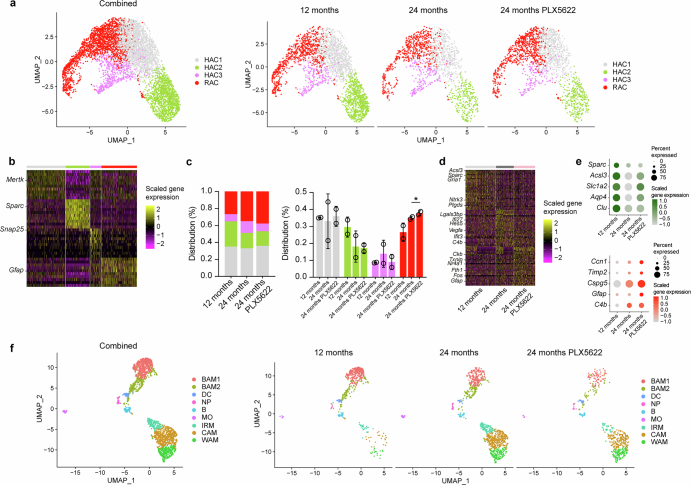
scRNA-seq reveals aging-related astrocyte and peripheral myeloid cell signatures upon PLX5622 treatment. **a**, UMAP visualization of fixed astrocytes sorted from adult (12-month-old), aged (24-month-old) and PLX5622-treated aged mouse brains and analyzed by scRNA-seq. Combined (left, 5,965 cells) and separate (right) visualizations of cells from adult (2,621 cells), aged (1,527 cells) and PLX5622-treated aged (1,817 cells) brains are displayed. **b**, Heatmap of cluster-specific genes. The color scale is based on a z-score distribution from −2 (purple) to 2 (yellow). **c**, Distribution of different cluster frequencies among all astrocytes and compositional comparison between samples (each circle represents one reaction; *n* = 2 reactions per group with 2 mice per reaction). Asterisks indicate significant differences identified by scCODA. Data are presented as the mean ± s.d. **d**, Heatmap of top 30 differentially expressed genes comparing astrocytes isolated from adult, aged and PLX5622-treated aged brains across all clusters as identified in **a**. **e**, Dot plot expression visualization of selected genes implicated in astrocyte homeostasis (HOM) and reactivity (RAC) across all astrocyte clusters. **f**, UMAP visualization of fixed border-associated cells sorted from adult, aged and PLX5622-treated aged mouse brains and analyzed by scRNA-seq. Combined (left, 5,965 cells) and separate (right) visualizations of cells from adult (2,621 cells), aged (1,527 cells) and PLX5622-treated aged (1,817 cells) brains are displayed. BAM, border-associated macrophages; DC, dendritic cells; NP, neutrophils; B, B cells; MO, monocytes.

When focusing on aging-related transcriptional changes in oligodendrocytes and astrocytes from our fixed single-cell RNA profiling, we found less prominent changes in the composition of aging-related oligodendrocyte states after PLX5622 treatment. However, when we compared oligodendrocytes from PLX5622-treated aged mice with untreated aged mice, there was an enrichment of upregulated genes related to protein folding, DNA damage and cell stress (Extended Data Fig. 6 and Supplementary Data 3). Moreover, we detected an increased frequency of reactive astrocytes in aged brains, which was further elevated by PLX5622 treatment. Reactive astrocytes showed signatures overlapping with neuroinflammatory and putative neurotoxic states previously identified in disease, neuroinflammation and aging^28–30^ (Extended Data Fig. 7a–e). We did not find a compensatory increase in peripheral myeloid cell frequency (dendritic cells, monocytes and neutrophils) upon PLX5622 treatment in brains from aged mice (Extended Data Fig. 7f).

Thus, PLX5622 treatment or CX3CR1 deficiency in aged mice impairs homeostatic microglia functions, resulting in a corresponding rise in pro-inflammatory microglia, changes in myelin integrity, degeneration of axons and deterioration in visual function.

### CD8^+^ T cells exacerbate axon loss in aged mice after PLX5622

**Fig. 4:**
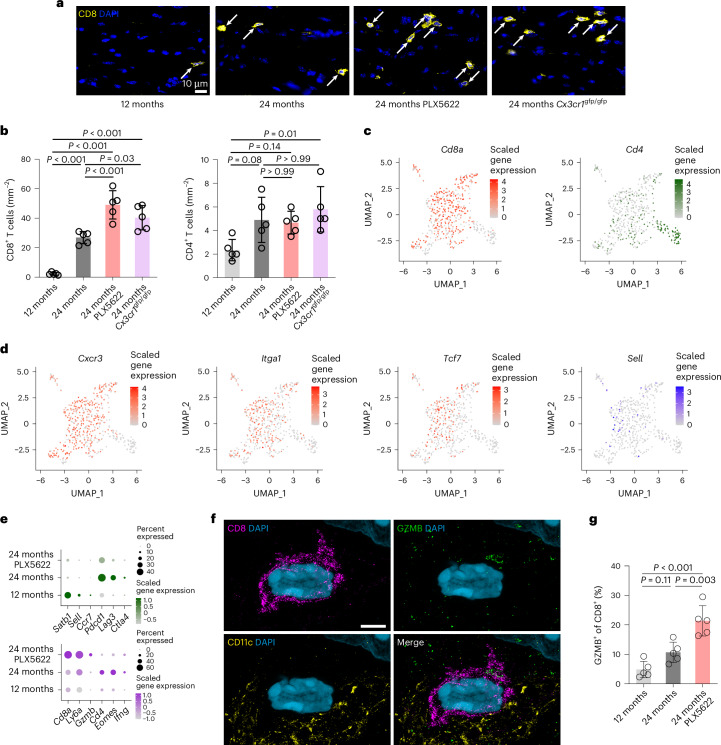
Maladaptive microglia activation promotes recruitment of CD8^+^ T cells in aged white matter. **a**, Immunofluorescence detection of CD8^+^ T cells (arrows) in longitudinal optic nerve section from adult (12-month-old), aged (24-month-old), aged PLX5622-treated and aged *Cx3cr1*^gfp/gfp^ mice. Scale bar, 10 μm. **b**, Quantification of CD8^+^ (left) and CD4^+^ (right) T cells (each circle represents the mean value of one mouse; *n* = 5 mice per group, CD8—*F*(3, 16) = 46.3, *P* < 0.001; CD4—*F*(3, 16) = 4.765, *P* = 0.01). **c**, UMAP visualizations of T cells (543 cells as annotated in Supplementary Fig. 5b) showing the expression of *Cd8a* (left) or *Cd4* (right). **d**, UMAP visualizations of T cells showing the expression of selected marker genes that indicate tissue recruitment, residency and memory function (red) or egress (blue). **e**, Dot plot expression visualization of selected genes implicated in central memory function and suppression of T cell activation (top) or cytotoxic effector and Th1 polarization (bottom) for T cells from adult, aged and PLX5622-treated aged brains. The color scales are based on *z-*score distributions. **f**, Super-resolution fluorescence detection of CD8, GZMB and CD11c in the optic nerves of an aged mouse. CD8^+^ T cells often contact CD11c^+^ microglia and contain GZMB^+^ granules (arrow). Scale bar, 10 µm expanded and 2.5 µm unexpanded. **g**, Quantification of GZMB^+^ cytotoxic T cells (percentage of CD8^+^) in optic nerves of adult, aged and PLX5622-treated aged mice (*n* = 5 mice per group, *F*(2, 12) = 22.65, *P* < 0.001). All statistical comparisons were performed with one-way ANOVA with Bonferroni’s multiple comparison tests. Data are presented as the mean ± s.d. Source data

**Extended Data Fig. 8:**
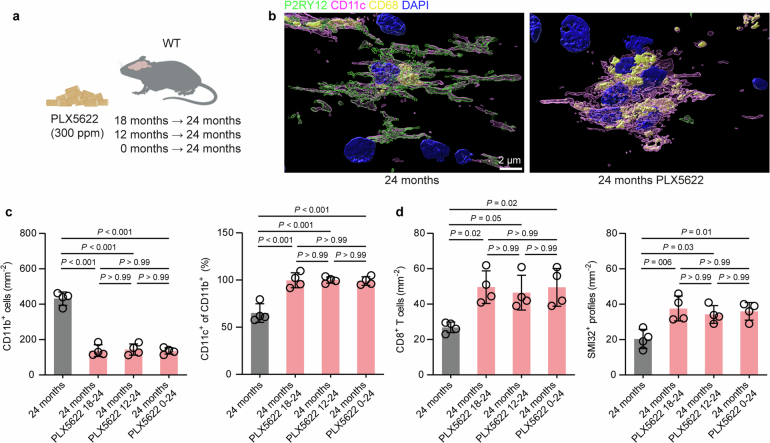
Different PLX5622 treatment durations similarly aggravate aging-related neuroinflammation and axon damage. **a**, Schematic experimental design. Panel **a** is created with BioRender.com. **b**, Immunofluorescence detection and IMARIS Z-stack surface rendering of P2RY12 in combination with CD11c and CD68 in optic nerves of aged and PLX5622-treated aged mice. CD11c^+^ microglia resisting PLX5622 depletion typically show lack of P2RY12 and strong reactivity for the lysosomal marker CD68. Scale bar, 5 µm. **c**, Immunofluorescence-based quantification of CD11b^+^ (left) microglia and CD11c^+^ (right) microglia (% of CD11b^+^) in longitudinal optic nerve sections from aged and aged PLX5622-treated mice with different treatment durations (each circle represents the mean value of one mouse; *n* = 4 mice per group, one-way ANOVA with Bonferroni’s multiple comparisons test, CD11b—*F*(3, 12) = 88.77, *P* < 0.001; CD11c—*F*(3, 12) = 24.93, *P* < 0.001). **d**, Immunofluorescence-based quantification of CD8^+^ (left) T cells and SMI32^+^ (right) axonal spheroids in longitudinal optic nerve sections from aged and aged PLX5622-treated mice with different treatment durations (*n* = 4 mice per group, one-way ANOVA with Bonferroni’s multiple comparisons test, CD8—*F*(3, 12) = 6.8, *P* = 0.008; SMI32—*F*(3, 15) = 7.76, *P* = 0.004). Data are presented as the mean ± s.d. Source data

To explore how increased pro-inflammatory activation and dysfunction result in aggravated loss of myelinated fibers, we characterized adaptive immune reactions, known to contribute to aging-related white matter damage^22,23^. PLX5622 treatment or CX3CR1 deficiency resulted in an increase in parenchymal CD8^+^ T cell numbers in the optic nerves of aged mice, while the generally lower CD4^+^ T cell numbers were unchanged (Fig. 4a,b and Supplementary Fig. 6a,b). Moreover, CD8^+^ T cells were more frequently found in proximity to CD11c^+^ microglia in aged PLX5622-treated or *Cx3cr*^gfp/gfp^ mice (Supplementary Fig. 7a). The increase in CD8^+^ T cell numbers and axon damage was also found when we applied different PLX5622 treatment regimens to deplete microglia, from 18 to 24 months, from 12 to 24 months or continuously from 0 to 24 months (Extended Data Fig. 8a–d). We analyzed our fixed scRNA-seq dataset and found that the T cell cluster showed a shift from the expression of CD4^+^ Th1 cell markers toward CD8^+^ T cell markers in aged PLX5622-treated mice (Fig. 4c–e). These CD8^+^ T cells closely resembled TRM T cells, maintaining the expression of effector and retention molecules but lacking markers of tissue egress. Notably, the expression of several markers of cytotoxic effector function (*Cd8a*, *Ly6a* and *Gzmb*) was upregulated in T cells after partial microglia depletion, while the expression of inhibitory receptors (*Pdcd1*, *Lag3* and *Ctla4*) was downregulated. Super-resolution fluorescence microscopy confirmed an increased expression of the cytotoxic effector protease granzyme B (GZMB) by CD8^+^ T cells after PLX5622 treatment in aged optic nerves (Fig. 4f,g and Supplementary Fig. 7b). Both PLX5622 treatment and CX3CR1 deficiency also resulted in an increased frequency of CD8^+^ T cells near SMI32^+^ axonal profiles, particularly of those immunoreactive for GZMB (Supplementary Fig. 7c,d). SMI32^+^ axonal spheroids were frequently (~70%) ensheathed by processes positive for SERPINA3N, a marker for reactive oligodendrocytes in aging^23^ (Supplementary Fig. 7e). Notably, PLX5622 treatment or CX3CR1 deficiency did not result in increased microglial activation, CD8^+^ T cell accumulation or axonal spheroid formation in adult mice (Supplementary Fig. 8).

**Fig. 5:**
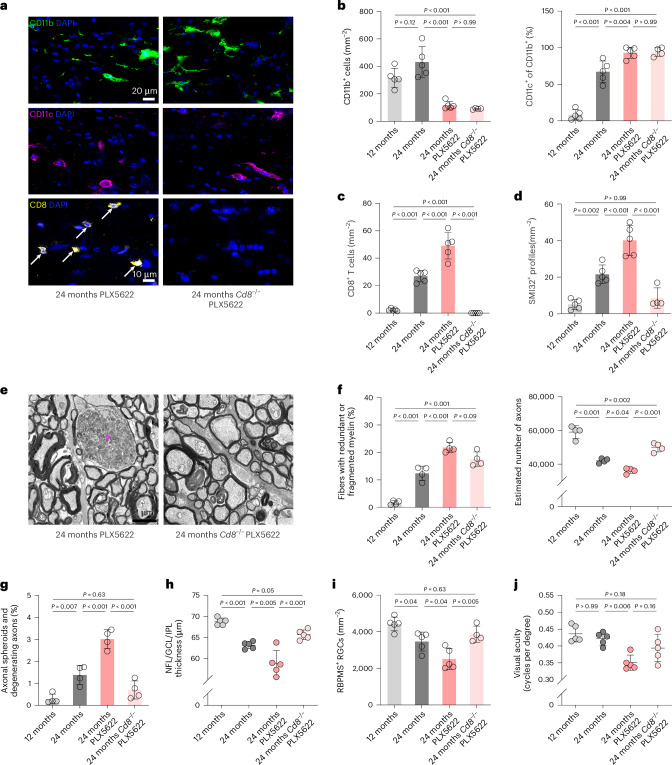
CD8^+^ T cells drive aggravated axon degeneration in PLX5622-treated aged mice. **a**–**c**, Immunofluorescence detection of CD11b (top), CD11c (middle) and CD8 (bottom, arrows; **a**) and quantification of CD11b^+^ (left) microglia, CD11c^+^ (right) microglia (percentage of CD11b^+^; **b**) and CD8^+^ T cells in longitudinal optic nerve sections from adult (12-month-old), aged (24-month-old), aged PLX5622-treated and aged PLX5622-treated *Cd8*^−/−^ mice (**c**; each circle represents the mean value of one mouse; *n* = 4–5 mice per group, CD11b—*F*(3, 15) = 26.0, *P* < 0.001; CD11c—*F*(3, 15) = 85.04, *P* < 0.001; CD8—*F*(3, 16) = 96.61, *P* < 0.001). Scale bars, 20 μm (top) and 10 µm (bottom). **d**, Immunofluorescence-based quantification of SMI32^+^ axonal spheroids (*n* = 4–5 mice per group, *F*(3, 15) = 36.58, *P* < 0.001). **e**, Representative electron micrographs of optic nerve cross-sections from aged PLX5622-treated and aged PLX5622-treated *Cd8*^−/−^ mice. The circle indicates an axonal spheroid. Scale bar, 2 μm. **f**, Electron microscopy-based quantification of fibers ensheathed with redundant or fragmented myelin (left) and estimation of total axonal numbers in the optic nerves (right; *n* = 4 mice per group, *F*(3, 12) = 67.99, *P* < 0.001). **g**, Quantification of fibers showing axonal spheroid formation or axon degeneration (right; *n* = 4 mice per group, *F*(3, 12) = 38.8, *P* < 0.001). **h**, OCT analysis of the innermost retinal composite layer (NFL/GCL/IPL) thickness in peripapillary circle scans (*n* = 5 mice per group, *F*(3, 16) = 28.93, *P* < 0.001). **i**, Quantification of RBPMS^+^ retinal ganglion cells (right; *n* = 4–5 mice per group, *F*(3, 15) = 13.65, *P* < 0.001). **j**, Automated optokinetic response analysis of visual acuity (cycles per degree) shows a decline of visual acuity in PLX5622-treated aged but not PLX5622-treated aged *Cd8*^−/−^ mice (*F*(3, 16) = 9.068, *P* < 0.001). All statistical comparisons were performed with one-way ANOVA with Bonferroni’s multiple comparison tests. Data are presented as the mean ± s.d. Source data

To determine whether the exacerbated neurodegeneration after PLX5622 treatment is mediated by the increased accumulation of CD8^+^ T cells, we treated *Cd8*^−/−^ mice from 18 to 24 months with PLX5622. The effectiveness of depletion, the predominant CD11c expression on remaining microglia and the higher occurrence of axons wrapped in excessive or fragmented myelin were similar between PLX5622-treated aged WT and *Cd8*^−/−^ mice (Fig. 5a–f). However, lack of CD8^+^ T cells in PLX5622-treated aged mice mitigated the formation of axonal spheroids, axon degeneration, loss of retinal ganglion cells and inner retinal thinning (Fig. 5c–i). This was in line with improved maintenance of visual acuity, as determined by OptoDrum analysis (Fig. 5j).

Thus, CD8^+^ T cells exacerbate axon degeneration in aged mice following PLX5622 treatment.

### MERFISH identifies *Cxcl10* near T cells in aged optic nerves

**Fig. 6:**
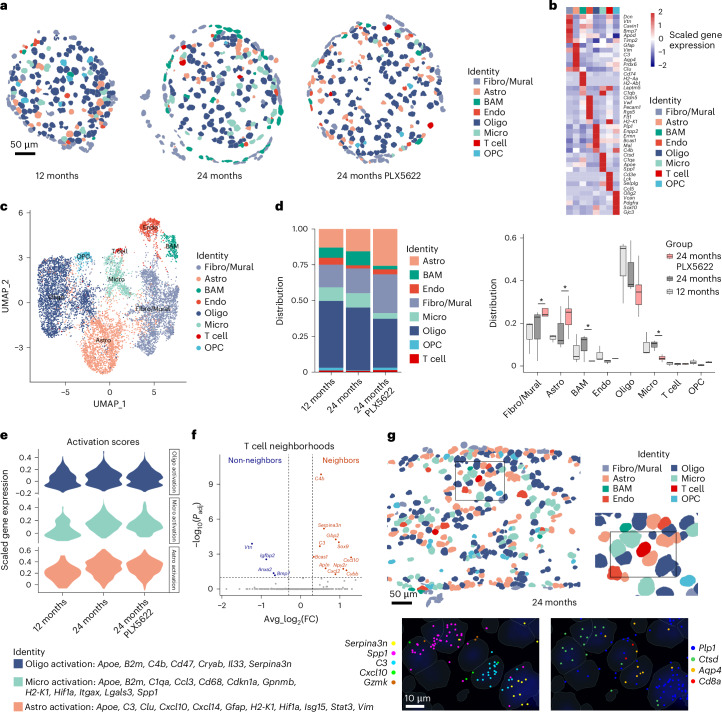
Spatially resolved single-cell transcriptomics of aged optic nerves upon PLX5622 treatment. **a**, Representative spatial plots of single-cell transcriptional profiles of optic nerve cross-sections from adult (12-month-old), aged (24-month-old) and PLX5622-treated aged mice measured using MERFISH. Cells are colored according to the major cell type clusters. Scale bar, 50 µm. **b**, Heatmap of cluster-specific gene expression levels. The color scale is based on a *z-*score distribution from −2 (navy blue) to 2 (red). **c**, UMAP visualization of all segmented cells from adult (576 cells), aged (2,360 cells) and PLX5622-treated aged (6,882 cells) optic nerves (*n* = 1–3 sections per nerve from three mice per group). **d**, Distribution of different cluster frequencies among all cells and compositional comparison between groups based on optic nerve cross-sections (*n* = 1–3 sections per nerve from three mice per group). The box and whisker plot elements in the figure represent the following: centerline, median; box limits, upper and lower quartiles; whiskers, minimum to maximum values. Asterisks indicate significant differences identified by scCODA. **e**, Violin plot of the expression of gene module scores indicating activation of oligodendrocytes, microglia and astrocytes in the corresponding cell type clusters based on MERFISH. Curated lists of activation scores are indicated at the bottom. **f**, Volcano plot of differential gene expression between 50 nearest neighbors of T cells and non-neighboring cells. Bonferroni-adjusted *P* values from a two-sided Wilcoxon rank-sum test are shown. **g**, Representative spatial plot of single-cell transcriptional profiles of a longitudinal optic nerve section from an aged mouse measured using MERFISH. Scale bar, 50 µm. Higher magnification insets show cell type composition (top right), activation markers (bottom left) and cell type markers (bottom right) at subcellular transcript resolution. Scale bar, 10 µm. Fibro/Mural, fibroblasts/mural cells; Astro, astrocytes; BAM, border-associated macrophages; Endo, endothelial cells; Oligo, oligodendrocytes; Micro, microglia; OPC, oligodendrocyte precursor cells.

How do activated microglia orchestrate the recruitment of CD8^+^ T cells in aged white matter? We performed spatially resolved single-cell transcriptomics using MERFISH with a custom-designed 500-gene panel on transverse or longitudinal sections of optic nerves from adult, aged and PLX5622-treated aged mice. This approach allowed us to identify all major cell types in optic nerve parenchyma and meningeal structures and probe their expression of selected genes (Fig. 6a–c). Again, PLX5622 treatment in aged nerves was reflected by partial depletion of microglia and border-associated macrophages (Fig. 6d). Moreover, a higher contribution of fibroblasts/mural cells and astrocytes to the total cell distribution and a trend toward lower number of oligodendrocytes were detected in PLX5622-treated compared with nontreated aged mice. In comparison, oligodendrocyte precursor cells and T cells were rare on cross-sections, and we did not identify substantial changes in their contribution to the overall cell composition. However, when looking for individual parenchymal T cells per section, we again detected the highest number in aged PLX5622-treated animals (adult, 1.0 ± 1.0; aged, 3.33 ± 0.58; aged PLX, 4.67 ± 1.53 T cells per section).

Next, we compared gene module scores indicative of glial activation in our MERFISH data using a curated list of transcripts based on previous studies^31,32^ and our own single-cell transcriptomic characterization. Aging resulted in higher activation scores for oligodendrocytes, astrocytes and microglia (Fig. 6e and Supplementary Fig. 9a). Consistent with our scRNA-seq analysis, PLX5622 treatment further elevated the expression of microglial activation markers while less prominently and distinctly affecting homeostatic and reactive marker genes for oligodendrocytes. Moreover, in addition to their increased number, astrocytes showed higher expression of activation markers.

We used the ability of MERFISH to study spatial expression profiles and cell neighborhoods at subcellular transcript resolution. Focusing on T cells, we confirmed that most of the T cells resembled CD8^+^ TRM and often expressed cytotoxic effector molecules (Supplementary Fig. 9b). We observed that T cells and microglia showed a significant neighborhood score for transcripts expressed by activated macroglia, including *Cxcl10* and *Serpina3n*, which we mostly detected in reactive astrocytes and oligodendrocytes, respectively (Fig. 6f and Supplementary Fig. 9c). Indeed, CD8^+^ T cells were preferentially detected in proximity to reactive oligodendrocytes, microglia and astrocytes (Fig. 6g). Moreover, MERFISH and immunofluorescence showed that PLX5622 treatment results in higher expression of pro-inflammatory activation markers and corroborated distinct WAM states (Supplementary Fig. 9d,e).

### CXCL10–CXCR3 signaling recruits CD8^+^ T cells in aging nerves

**Extended Data Fig. 9:**
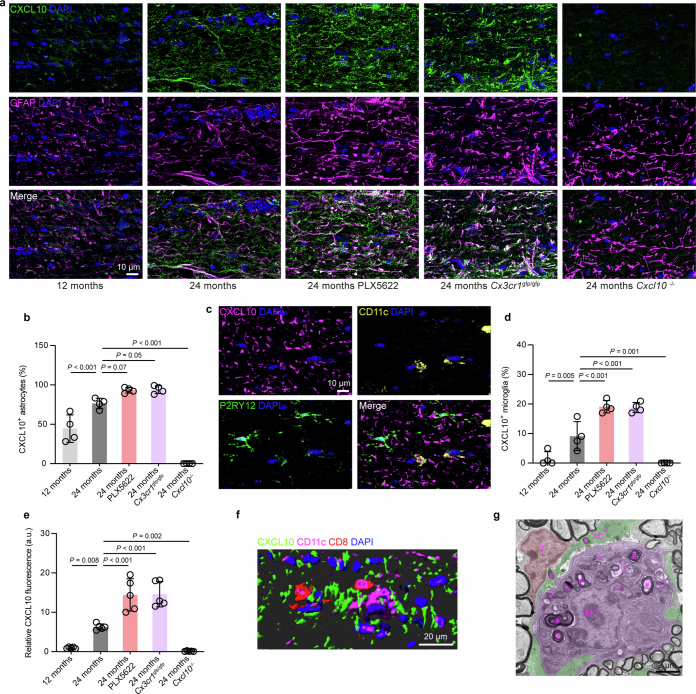
PLX5622 treatment or CX3CR1 deficiency enhance aging-related expression of CXCL10. **a**,**b**, Immunofluorescence detection (**a**) and quantification (**b**) of CXCL10^+^ astrocytes (% of GFAP^+^) in longitudinal optic nerve sections from adult (12-month-old), aged (24-month-old), aged PLX5622-treated and aged *Cx3cr1*^gfp/gfp^ mice (each circle represents the mean value of one mouse; *n* = 4 mice per group, one-way ANOVA with Bonferroni’s multiple comparisons test, top—*F*(4, 15) = 81.85, *P* < 0.001). Scale bar, 10 μm. **c**,**d**, Immunofluorescence detection (**c**) and quantification (**d**) of CXCL10^+^ microglia (% of P2RY12^+^ or CD11c^+^) in longitudinal optic nerve sections from adult, aged, aged PLX5622-treated and aged *Cx3cr1*^gfp/gfp^ mice (*n* = 4 mice per group, one-way ANOVA with Bonferroni’s multiple comparisons test, top—*F*(4, 15) = 45.00, *P* < 0.001). Scale bar, 10 μm. **e**, Quantification of CXCL10 fluorescence in optic nerves by thresholding analysis (*n* = 5 mice per group, one-way ANOVA with Bonferroni’s multiple comparisons test, *F*(4, 25) = 43.83, *P* = 0.001). **f**, Immunofluorescence detection and IMARIS Z-stack surface rendering of CXCL10 in combination with CD11c and CD8 in the optic nerve from a PLX5622-treated aged mouse. An activated microglial cell is found in proximity to a CXCL10^+^ putative astrocyte and a CD8^+^ T cell. Scale bar, 20 µm. **g**, Electron micrograph of the optic nerve from an aged PLX5622-treated mouse; a microglial cell (MG) containing intracellular myelin fragments (hashtags), small lipid droplets (open arrowheads) and lysosomal storage material (asterisks) is found in proximity to a fibrous astrocyte (AC) and a putative T cell (T). Scale bar, 2 µm. Data are presented as the mean ± s.d. Source data

The proximity of T cells and microglia to *Cxcl10*-expressing cells caught our attention, as CXCL10 is a significant chemoattractant for T cells^33^. Additionally, our analyses showed that its receptor, CXCR3, is expressed on nearly all CD8^+^ T cells in the aged brain parenchyma^22^ (Fig. 4d). Our single-cell and spatial transcriptomic analysis indicated the strongest *Cxcl10* transcription in reactive astrocytes and microglia (and T cells themselves). CellChat analysis of our fixed scRNA-seq data revealed intricate interactions between various reactive glia and T cells, including *Cxcl10*–*Cxcr3* signaling, and we confirmed the increased expression of *Cxcl10* in PLX5622-treated aged optic nerves by qRT–PCR (Supplementary Fig. 10a–c). Using immunofluorescence, we validated CXCL10 protein expression by GFAP^+^ astrocytes and CD11b^+^ microglia in optic nerves of adult and aged mice (Extended Data Fig. 9a). Consistent with the increased recruitment of CD8^+^ T cells, PLX5622 treatment or CX3CR1 deficiency increased the expression of CXCL10 and GFAP reactivity in astrocytes from aged mice (Extended Data Fig. 9a,b,e). Similarly, CXCL10 immunoreactivity was increased in CD11c^+^ microglia after PLX5622 or CX3CR1 deficiency in aged mice, while homeostatic P2RY12^+^ microglia rarely expressed CXCL10 (Extended Data Fig. 9c–e). Reminiscent of our MERFISH data, CD8^+^ T cells were frequently detected in association with CXCL10^+^ astrocytes and activated CD11c^+^ microglia (Extended Data Fig. 9f,g and Supplementary Fig. 10d,e).

**Extended Data Fig. 10:**
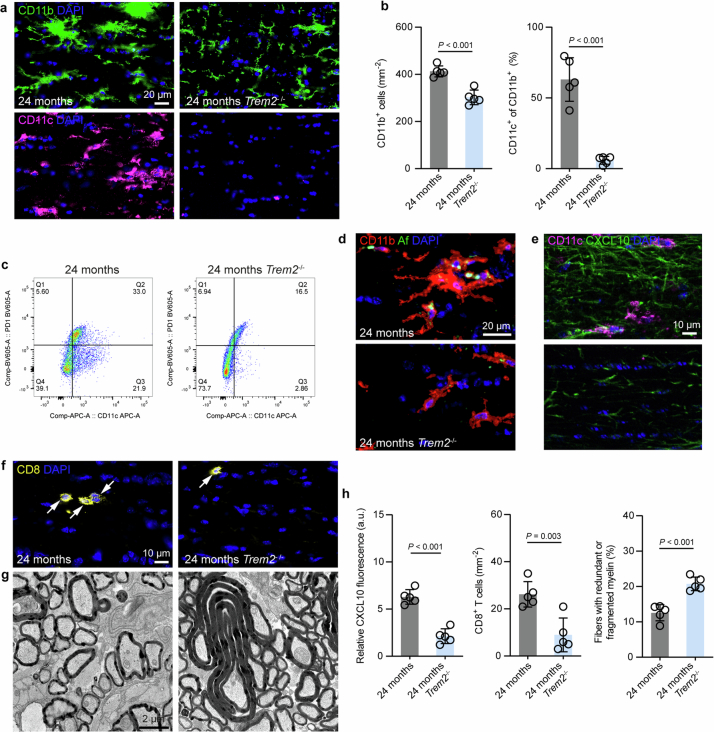
TREM2 deficiency mitigates aging-related neuroinflammation, CXCL10 expression and CD8^+^ T cell recruitment. **a**,**b**, Immunofluorescence detection (**a**) and quantification (**b**) of CD11b^+^ (top) or CD11c^+^ (bottom) microglia (% of CD11b^+^) in longitudinal optic nerve sections from aged (24-month-old) and aged *Trem2*^−/−^ mice (each circle represents the mean value of one mouse; *n* = 5 mice per group, unpaired two-sided Student’s *t*-test, left: *t* = 6.407, df = 8, right: *t* = 8.171, df = 8). Scale bar, 20 μm. **c**, Representative flow cytometry plots for gating of single, viable CD45^low^Siglec-H^+^ microglia from aged and aged *Trem2*^−/−^ brains. Formation of both CD11c^+^PD-1^+^ (WAM1) and CD11c^+^PD-1^−^ (WAM2) microglia depends on TREM2 signaling. **d**, Immunofluorescence detection of CD11b^+^ microglia and autofluorescent storage material (Af) in optic nerves from aged and aged *Trem2*^−/−^ mice. Scale bar, 20 µm. **e**,**f**, Immunofluorescence detection of CXCL10 in combination with CD11c (**e**) and CD8^+^ T cells (**f**) in longitudinal optic nerve sections from aged and aged *Trem2*^−/−^ mice. Scale bar, 10 μm. **g**, Representative electron micrographs of optic nerve cross-sections from aged and aged *Trem2*^−/−^ mice. Scale bar, 2 μm. **h**, Quantification of CXCL10 fluorescence, CD8^+^ T cells and fibers ensheathed with redundant or fragmented myelin in optic nerves from aged and aged *Trem2*^−/−^ mice (*n* = 5 mice per group, unpaired two-sided Student’s *t*-test, left: *t* = 8.503, df = 8, middle: *t* = 4.281, df = 8, right: *t* = 6.081, df = 8). Data are presented as the mean ± s.d. Source data

Our findings indicated that microglia activation in aged white matter triggers a response in astrocytes, thereby promoting enhanced recruitment of CD8^+^ T cells. To assess this, we took advantage of aged *Trem2*^−/−^ mice to lock microglia in a homeostatic state^16,34^. As shown previously, absence of TREM2 prevented the aging-related increase in microglial numbers and their CD11c expression (Extended Data Fig. 10a–c). Immunofluorescence revealed less autofluorescent storage material in microglia and decreased expression of CXCL10 in optic nerves from aged *Trem2*^−/−^ compared with WT mice (Extended Data Fig. 10d–h). Moreover, the accumulation of CD8^+^ T cells was attenuated, while the frequency of fibers ensheathed with aberrant myelin was increased by TREM2 deficiency in aging.

**Fig. 7:**
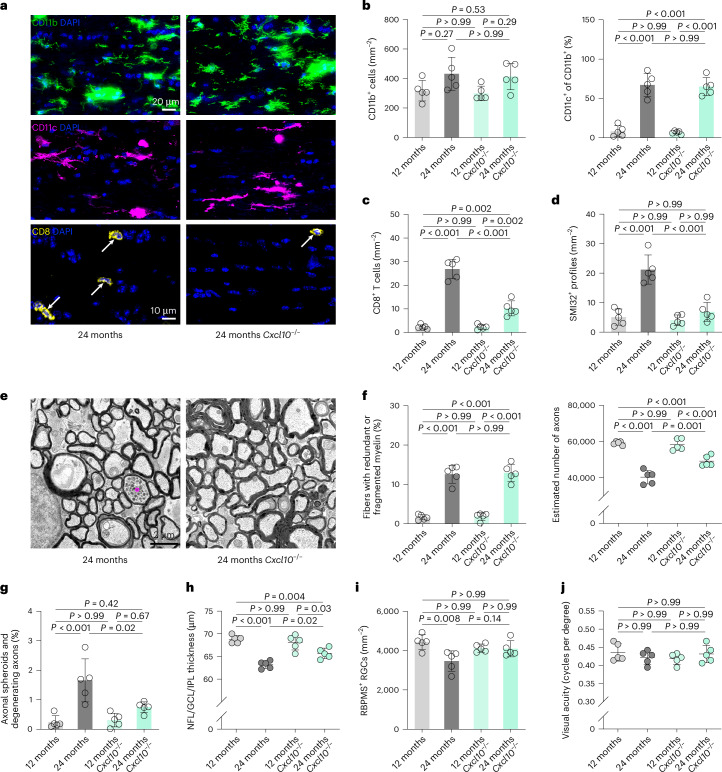
CXCL10 mediates CD8^+^ T cell recruitment and axon degeneration in aged optic nerves. **a**–**c**, Immunofluorescence detection of CD11b (top), CD11c (middle) and CD8 (bottom, arrows; **a**) and quantification of CD11b^+^ (left) microglia, CD11c^+^ (right) microglia (percentage of CD11b^+^; **b**) and CD8^+^ T cells in longitudinal optic nerve sections from adult (12-month-old), adult Cxcl10^−/−^, aged (24-month-old), aged *Cxcl10*^−/−^ mice (**c**; each circle represents the mean value of one mouse; *n* = 5 mice per group, CD11b—*F*(3, 16) = 3.193, *P* = 0.05; CD11c—*F*(3, 16) = 58.10, *P* < 0.001; CD8—*F*(3, 16) = 92.30, *P* < 0.001). Scale bars, 20 μm (top) and 10 µm (bottom). **d**, Immunofluorescence-based quantification of SMI32^+^ axonal spheroids (*n* = 5 mice per group, *F*(3, 16) = 28.32, *P* < 0.001). **e**, Representative electron micrographs of optic nerve cross-sections from aged and aged *Cxcl10*^−/−^ mice. The circle indicates an axonal spheroid. Scale bar, 2 μm. **f**, Electron microscopy-based quantification of fibers ensheathed with redundant or fragmented myelin (left) and estimation of total axonal numbers in the optic nerves (right; *n* = 5 mice per group, *F*(3, 16) = 70.22, *P* < 0.001). **g**, Quantification of fibers showing axonal spheroid formation or axon degeneration (*n* = 4 mice per group, *F*(2, 36) = 12.76, *P* < 0.001). **h**, OCT analysis of the innermost retinal composite layer (NFL/GCL/IPL) thickness in peripapillary circle scans (*n* = 5 mice per group, *F*(3, 16) = 23.79, *P* < 0.001). **i**, Quantification of RBPMS^+^ retinal ganglion cells (*n* = 5 mice per group, *F*(3, 16) = 5.225, *P* = 0.01). **j**, Automated optokinetic response analysis of visual acuity (cycles per degree; n = 5 mice per group, *F*(3, 16) = 0.7716, *P* = 0.53). All statistical comparisons were performed with one-way ANOVA with Bonferroni’s multiple comparison tests. Data are presented as the mean ± s.d. Source data

To directly investigate whether CXCL10 is involved in the recruitment of CD8^+^ T cells to aged white matter, we analyzed adult and aged *Cxcl10*^−/−^ mice. The number of microglia, their expression of CD11c and the increased number of axons ensheathed by redundant or fragmented myelin were comparable for aged WT and *Cxcl10*^−/−^ mice (Fig. 7a–f). However, lack of CXCL10 in aged mice attenuated the accumulation of CD8^+^ T cells, formation of axonal spheroids, axon degeneration, retinal thinning and loss of retinal ganglion cells (Fig. 7c–j). Adult *Cxcl10*^−/−^ mice demonstrated no obvious differences compared with WT mice, arguing for an effect on aging-related neuroinflammation rather than general white matter architecture.

**Fig. 8:**
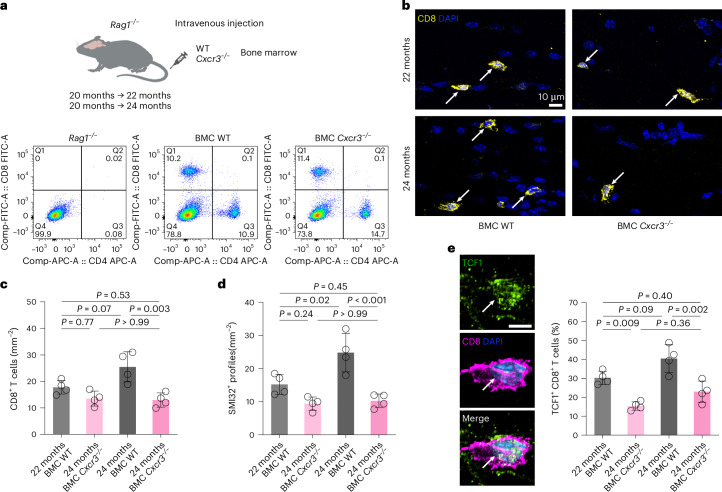
Hematopoietic CXCR3 deficiency attenuates persistence of T cells in optic nerves of aged mice. **a**, Schematic experimental design (top) and flow cytometry (bottom) of aged (24-month-old) splenocytes from *Rag1*^−/−^ mice with or without transfer of bone marrow from WT (BMC WT) or *Cxcr3*^−/−^ (BMC *Cxcr3*^−/−^) donors for presence of CD8^+^ and CD4^+^ T cells. Panel **a** is created with BioRender.com. **b**, Immunofluorescence detection of CD8^+^ T cells (arrows) in longitudinal optic nerve sections from 22- and 24-month-old BMC WT and BMC *Cxcr3*^−/−^ mice. Scale bar, 10 µm. **c**,**d**, Quantification of CD8^+^ T cells (**c**) and SMI32^+^ axonal spheroids (**d**; each circle represents the mean value of one mouse; *n* = 4 mice per group, CD8—*F*(3, 12) = 9.861, *P* = 0.001; SMI32—*F*(3, 12) = 15.59, *P* < 0.001). **e**, Immunofluorescence detection (left) and quantification (right) of TCF1 in CD8^+^ T cells in longitudinal optic nerve sections from 22- to 24-month-old BMC WT and BMC *Cxcr3*^−/−^ mice (*n* = 4 mice per group, *F*(3, 12) = 17.4, *P* < 0.001). All statistical comparisons were performed with one-way ANOVA with Bonferroni’s multiple comparison tests. Data are presented as the mean ± s.d. BMC, bone marrow chimeras. Source data

Because our data showed that CXCR3, the known receptor for CXCL10, is expressed on nearly all CD8^+^ T cells in the aged brain, we explored its role in aging-related accumulation and localization of CD8^+^ T cells to the optic nerve. We transferred bone marrow from wild-type (WT) or *Cxcr3*^−/−^ donor mice into 20-month-old *Rag1*-deficient mice and analyzed the recipients at 22 or 24 months of age. Reconstitution with WT bone marrow restored the peripheral T cell compartment and the gradual recruitment of CD8^+^ T cells into the white matter of aging mice (Fig. 8a–c). In contrast, reconstitution with *Cxcr3*^−/−^ bone marrow restored peripheral T cells and their early recruitment to the white matter but resulted in a substantially reduced accumulation of CD8^+^ T cells at 24 months of age. A corresponding attenuation of axonal spheroid formation in aged *Cxcr3*^−/−^ bone marrow chimeras compared with WT chimeras was detected (Fig. 8d). These results suggest that CXCR3 signaling fosters the long-term persistence and tissue residency program of CD8^+^ CNS-associated T cells. We therefore investigated the expression of TCF1, a transcription factor central to stemness and CNS persistence^35^, in CD8^+^ T cells from optic nerves of the bone marrow chimeric mice. At both investigated time points, absence of CXCR3 decreased the percentage of TCF1^+^ CD8^+^ T cells (Fig. 8e).

## Discussion

Aging is associated with oligodendrocyte changes and white matter pathology, which includes focal formation of fragmented and redundant myelin, as well as axonal damage and degeneration. These alterations are accompanied by distinct microglial activation states and the low-grade accumulation of CD8^+^ T cells, which we corroborated in aged human brains. The precise impact of these neuroinflammatory responses on different aging-related pathologies remains incompletely understood. In this study, we used PLX5622 to chronically ablate CSF-1R-dependent myeloid cells from the CNS, which resulted in the preferential depletion of homeostatic microglia while preserving and enforcing various states of WAM. Additionally, we impaired microglial function by CX3CR1 deficiency, which also resulted in exacerbated microglial activation. As a result, there was an increased accumulation of CD8^+^ T cells and damage to myelinated axons, resulting in aggravated neurodegeneration and accelerated behavioral decline. scRNA-seq indicated that most of these T cells were CD8^+^ TRM, which showed higher expression of cytotoxic molecules (that is, GZMB) previously linked to aging-related axon degeneration and behavioral decline^22^. Using spatial transcriptomics, we identified the niche of CD8^+^ TRM, comprising reactive astrocytes and microglia with elevated *Cxcl10* expression. Mechanistically, we demonstrated that the CXCL10–CXCR3 axis is involved in attracting and retaining CD8^+^ T cells in the brain, thereby promoting their harmful impact on aged white matter. Our findings suggest that maladaptive white matter-associated microglial states can promote immune responses in aging and highlight potential targets to reduce their detrimental effects.

These results raise several questions. The first pertains to the role of microglia in the aged white matter. While we cannot rule out direct effects of PLX5622 treatment or CX3CR1 deficiency on other cells (for example, neurons, astrocytes and peripheral immune cells), the functional outcomes of PLX5622 treatment on the pathology of myelinated axons could be explained by a combination of loss-of-function and gain-of-toxic-function effects in microglia. Loss-of-function in microglia following PLX5622 treatment may arise from the depletion of protective microglia states, resulting in insufficient support of neurons and oligodendroglia. Recent evidence suggests that microglia have a role in maintaining long-term myelin integrity by preventing its overproduction and subsequent degeneration^36^. This process appears to be regulated by transforming growth factor beta (TGFβ) released from microglia, which acts on transforming growth factor beta receptor 1 (TGFβR1)-expressing oligodendrocytes. Along these lines, recent studies show that the absence of microglia or dysbalanced CSF-1R signaling leads to age-associated brain pathology and myelin aberrations and that transplantation of intact microglia, whether of mouse or human origin, largely prevents this pathology^37–39^. Additionally, PLX5622 treatment also seems to induce toxic functions of microglia in aged white matter. The reduction in microglia numbers leads to increased stress and exhaustion in the remaining populations, potentially causing them to become more pro-inflammatory, antigen-presenting and senescent with aging.

Our findings show that chronic PLX5622 treatment is linked to increased recruitment of CD8^+^ T cells and greater pathology of myelinated axons in aging, which is alleviated in mice lacking CD8^+^ T cells. In line with earlier research, the majority of these CD8^+^ T cells were identified as TRM, raising the question about their role in aging^22,23,40^. CD8^+^ TRM cells function as sentinels within tissues, quickly alerting their environment upon encountering an antigen to establish immediate local protection during reinfection^41–43^. While these cells are crucial for defending against local reinfections, their presence is detrimental in autoimmune diseases, where they contribute to compartmentalized disease pathology^44–47^. However, the precise, multifaceted roles of these cells in aging and various neurodegenerative disorders, where they have also been observed, remain unclear because these conditions are not considered to be driven by autoimmunity^48–52^. One possibility is that brain damage, and in particular myelin perturbation, is linked to leakage of antigens to the periphery where lymphocyte priming occurs^53,54^. Alternatively, antigen-independent recruitment and local antigen encounter and activation might dictate memory T cell formation^22,55^.

Our transcriptomic approaches allowed for a comprehensive examination of the responses of various glial and immune cell populations to aging and PLX5622 treatment. However, the applied assays did not facilitate the profiling of T cell receptor sequences. Identifying the clonality and putative antigens recognized by these T cells remains a critical question for ongoing investigations. Additionally, there is a need for new tools to elucidate the functions of CD8^+^ TRM and specific WAM states in greater detail because these cells either exhibit poor viability or rapidly lose their unique transcriptional profiles in cell culture.

Once CD8^+^ T cells settle and reside in the brain, they may exert their maladaptive, detrimental function when (re-)activated by antigen-presenting cells and target structures^53,56^. In mice with distinct myelin gene defects, cytotoxic CD8^+^ TRM induce cytoskeletal alterations in oligodendrocytes and thereby drive the degeneration of axons persistently ensheathed with perturbed myelin^57^. The increased expression of cytolytic molecules by T cells and antigen presentation-related molecules by glial cells could indicate that such processes are promoted by PLX5622 treatment in aging. In addition, we found a close interaction of CD8^+^ T cells and microglia with stressed, aging-related oligodendrocyte states associated with axonal spheroids in aged optic nerves. However, indirect antigen-independent effects of CD8^+^ TRM cells are also possible. Activated CD8^+^ TRM cells are known to also act through cytokine secretion, predominantly interferon gamma (IFN-γ)^55,58^. This process can stimulate the surrounding tissue to express molecules crucial for broad-spectrum host defense, leading to the establishment of an ‘anti-viral state’. Supporting this notion, recent findings have identified IFN-responsive glial states in proximity to CD8^+^ T cells within the white matter, which can cause demyelination and loss of oligodendrocytes^23^.

Due to their important roles across various diseases, the development of new approaches to target CD8^+^ TRM is crucial. Here we show that CD8^+^ T cells are recruited to the aging white matter through CXCL10, an IFN-stimulated chemokine for CXCR3^+^ cells^33^. Using MERFISH and immunofluorescence, *Cxcl10* transcripts and CXCL10 protein expression were found in the niche of CD8^+^ T cells, primarily in astrocytes and, to a lesser extent, in microglia. How is CXCL10 expression induced? Our findings suggest that microglial activation may trigger CXCL10 expression in astrocytes. In TREM2-deficient mice, where microglia remain in a homeostatic state and fail to initiate the WAM response^16^, we observed reduced CXCL10 levels in both microglia and astrocytes. Moreover, treatment with PLX5622 or CX3CR1 deficiency, which depletes homeostatic microglia and promotes the accumulation of activated microglia, increased CXCL10 expression. Thus, a plausible scenario is that aging-related oligodendrocyte/myelin perturbation and subsequent microglial reactivity provoke an astrocytic response, resulting in elevated CXCL10 levels and recruitment of CD8^+^ T cells into the brain. Microglia–astrocyte interactions and the upregulation of CXCL10 are common features in various neurological disorders and aging^30,59–61^, associated with increased numbers of CD8^+^CXCR3^+^ TRM cells. Expansion of these cells has been observed not only within the brain parenchyma but also in the cerebrospinal fluid of conditions such as multiple sclerosis and Alzheimer’s disease^49,62^.

Although further research is required, these studies collectively suggest a role for CD8^+^ TRM cells across the spectrum of aging, neuroimmunological and neurodegenerative diseases. Targeting these inflammatory pathways common to such diseases holds promise for treating maladaptive immune responses effectively.

## Methods

### Animals

All animal experiments were approved by the governments of Lower Franconia, Würzburg, Germany and Upper Bavaria, Munich, Germany (AZ 55.2 DMS 2532-2-1, AZ 55.2 DMS 2532-2-399, AZ 55.2 DMS 2532-2-907, AZ 55.2 DMS 2532-2-1029 and AZ 55.2 DMS 2532-2-1191) and comply with the relevant ethical regulations. Mice were kept at the animal facility of the Centre for Experimental Molecular Medicine, University of Würzburg, or the German Center for Neurodegenerative Diseases (DZNE) in Munich under barrier conditions and at a constant cycle of 12 h in the light (<300 lux) and 12 h in the dark. Colonies were maintained at 20–24 °C and 40–60% humidity, with free access to food and water. All mice including WT, *Cx3cr1*^gfp/gfp^ (B6.129P2(Cg)-*Cx3cr1*^tm1Litt^/J)^63^, *Cd8*^−/−^ (B6.129S2-*Cd8a*^tm1Mak^/J)^64^, *Trem2*^−/−^ (B6.129P2-*Trem2*^tm1cln^/J)^65^, *Cxcl10* (B6.129S4*-Cxcl10*^tm1Adl^/J)^66^, *Rag1*^−/−^ (B6.129S7-*Rag1*^*tm1Mom*^/J)^67^ and *Cxcr3*^−/−^ (B6.129P2-*Cxcr3*^*tm1Dgen*^/J) were on a uniform C57BL/6J genetic background; they were bred, regularly backcrossed and aged in-house. Because there were no obvious differences between male and female mice in the analyses presented in the current study, mice of either sex were used for most experiments. For PLX5622 treatment and scRNA-seq experiments, male mice were used. Genotypes were determined by conventional PCR using isolated DNA from ear punch biopsies.

### PLX5622 treatment

PLX5622 (provided by Plexxikon) was prepared as a 300 ppm drug chow. This corresponds to a dose of ~54 mg PLX5622 per kg body weight when given ad libitum and was based on our previous long-term treatment approaches in which we observed efficient microglia depletion without obvious neurological side effects^57,68,69^. Control mice received normal chow without the pharmacological inhibitor. Mice were treated for 6, 12 or 24 months with daily monitoring concerning certain burden criteria and phenotypic abnormalities. The treatment started before obvious aging-related pathology in mice at 18, 12 or 0 months of age (by postpartum treatment of lactating mothers and continued postweaning treatment of pups).

### Histochemistry and immunofluorescence

Mice were killed with CO_2_ (according to the guidelines by the State Office of Health and Social Affairs Berlin), blood was removed by transcardial perfusion with PBS containing heparin and tissue was fixed by perfusion with 2% PFA in PBS. Tissue was collected, postfixed, dehydrated and processed as described previously^22^. Immunohistochemistry was performed on 10- or 30-μm‐thick longitudinal optic nerve and coronal brain sections. Sections were postfixed in 4% PFA in PBS or ice‐cold acetone for 10 min. Afterward, sections were blocked using 5% BSA in PBS containing 0.3% Triton X-100 and incubated overnight at 4 °C with an appropriate combination of up to three of the following antibodies: rat anti-CD11b (Bio-Rad Laboratories, MCA74G; 1:100); hamster anti-CD11c (Thermo Fisher Scientific, MA11C5; 1:100); mouse antineurofilament H nonphosphorylated, SMI32 (BioLegend, 801701; 1:1,000); rat anti‐CD8 (Bio‐Rad Laboratories, MCA609G; 1:500); goat anti-GZMB (R&D Systems, AF1865; 1:100); rabbit anti-GZMB (Abcam, ab4059; 1:100); rabbit anti-Laminin (Abcam, ab11575; 1:300); rabbit anti-P2RY12 (AnaSpec, 55043A; 1:300); rabbit anti-GAL3 (Novus Biologicals, NBP3-03252; 1:1,000); rat anti-GAL3 (BioLegend, 125402; 1:300); goat anti-CXCL10 (R&D Systems, AF-466-NA; 1:500) mouse anti-GFAP (Sigma-Aldrich, G3893; 1:1,000), rabbit anti-TCF-1 (Cell Signaling Technology, 2203; 1:100); goat anti-SERPINA3N (Bio-Techne, AF4709; 1:100 dilution). Immunoreactive profiles were visualized using fluorescently labeled (Dianova or Thermo Fisher Scientific; 1:300) secondary antibodies; nuclei were stained with DAPI (Sigma‐Aldrich). Fluorescence microscopic images were acquired using an Axio Imager M2 microscope (ZEISS) with ApoTome.2 structured illumination equipment, attached Axiocam cameras and corresponding software (ZEN v.2.3 blue edition) or an LSM900 confocal microscope (ZEISS) with an AiryScan2 detector. Autofluorescent lipofuscin-like lysosomal storage material was detected by imaging an unstained channel with high sensitivity at the 488 nm wavelength. Images were minimally processed (rotation, cropping and addition of symbols) to generate figures using Photoshop CS6 and Illustrator CS6 (Adobe Creative Suite 6). Z-stack surface rendering was performed using IMARIS v.9.7 (Bitplane). For quantification, immunoreactive profiles were counted in at least three nonadjacent tissue sections for each animal and related to the area of these sections using the cell counter plugin in Fiji/ImageJ v.1.51 (National Institutes of Health). The proximity of CD8^+^ T cells or GZMB^+^CD8^+^ T cells to SMI32^+^ axonal spheroids, CD11c^+^ microglia or CXCL10^+^ cells was defined by a distance of <10 µm of the closest fluorescent signals to each other. To quantify RGCs, perfusion-fixed eyes were enucleated, and specific markers of the inner retinal cell types were labeled in free-floating retina preparations. Fixed retinae were permeabilized by freezing in PBS containing 2% Triton X-100, thawed, washed and blocked for 1 h using 5% BSA and 5% donkey serum in PBS containing 2% Triton X-100. Retinae were incubated overnight on a rocker at 4 °C with guinea pig anti-RBPMS (Merck Millipore, ABN1376; 1:300) antibodies; immune reactions were visualized using fluorescently labeled (Dianova; 1:500) secondary antibodies, retinae were flat-mounted in Aqua-Poly/Mount and the total retinal area was measured. RGCs were quantified in three images of the middle retinal region per flat mount using the cell counter plugin in Fiji/ImageJ v.1.51 (National Institutes of Health).

### Electron microscopy

Mice were transcardially perfused, and the optic nerves were postfixed overnight in 4% PFA and 2% glutaraldehyde in cacodylate buffer. Nerves were osmicated and processed for light and electron microscopy; morphometric quantification of neuropathological alterations was performed as published previously^22^ using a LEO906 E electron microscope (ZEISS) and corresponding software iTEM v.5.1 (Soft Imaging System). At least ten regions of interest (corresponding to an area of around 5% and up to 3,000 axons per individual optic nerve) were analyzed per optic nerve per mouse. The percentages of axonal profiles enwrapped with aberrant myelin or showing signs of spheroid formation/degeneration were counted individually by their characteristic morphological features in electron micrographs and related to the number of all investigated axons per optic nerve per mouse. Axons were classified as ensheathed with redundant or fragmented myelin when their myelin sheath showed prominent outfoldings that remained continuous or were partially interrupted within the plane of the section. Axons without myelin or those with thin myelin (*g* ratio ≥ 0.85) were quantified and grouped together or shown separately. Axons showing accumulation of organelles and dense bodies and an increase in diameter were classified as spheroids, and those showing condensed or disrupted cytoplasm were classified as degenerating. Microglia were identified based on established ultrastructural characteristics and grouped into different categories according to their major defining features, including endoplasmic reticulum and mitochondria content, myelin fragments, lipid droplets, and lysosomal storage material. Images were processed (rotation, cropping, addition of symbols and pseudocolor) to generate figures using Photoshop CS6 and Illustrator CS6 (Adobe Creative Suite 6).

### Flow cytometry and cell sorting

Mice were killed with CO_2_ (according to the guidelines by the State Office of Health and Social Affairs Berlin), and blood was thoroughly removed by transcardial perfusion with PBS containing heparin. Brain hemispheres, including leptomeninges and choroid plexus, were dissected, collected in ice‐cold PBS and cut into small pieces according to previously published protocols^57^. Tissue was digested in 1 ml of Accutase (Merck Millipore) per sample at room temperature for 15 min and triturated through 70-μm cell strainers, which were rinsed with 10% fetal calf serum (FCS) in PBS. Cells were purified by a linear 40% Percoll (GE Healthcare) centrifugation step at 650*g* without brakes for 25 min, and the myelin top layer and supernatant were discarded. Mononuclear cells were resuspended in 1% BSA in PBS, and isolated cells were counted for each brain. Fc receptors were blocked for 15 min with rat anti‐CD16/32 (BD Biosciences, 553141; 1:200), and cells were washed and labeled with the following antibodies for 30 min at 4 °C: rat anti-CD45 PerCP/Cyanine5.5 (Miltenyi Biotec, 130-102-469; 1:100) and rat anti-Siglec-H PE (eBioscience, 12-0333-82; 1:100). Viable cells were identified by lack of DAPI stain (Biomol, ABD-22007). Cells were washed twice, single viable cells were gated and around 15,000 CD45^low^ Siglec-H^+^ cells were collected using a FACSAria III and corresponding software (FACSDiva, v.6; BD Biosciences). For further experiments, viable CD45^low^Siglec-H^+^ microglia were labeled with rat anti-CD11c APC (BioLegend, 117310; 1:100) and rat anti-PD1 BV605 (BioLegend, 135219; 1:100). Cells were washed twice; single viable cells were gated, and CD45^low^Siglec-H^+^ cells were analyzed using a FACSLyric (BD Biosciences) and FlowJo (v.10). For fixed RNA profiling, fresh tissue was directly chopped and fixed before dissociation (Supplementary Fig. 1) or snap-frozen in liquid nitrogen and stored at -80 °C before chopping and fixation (Supplementary Fig. 5). Tissue pieces were fixed with 2% PFA in PBS for 2 h on ice, digested in Accutase for 30 min at room temperature, triturated and purified as described above. Cells were washed and permeabilized with 1% BSA in PBS containing 0.2% Tween 20 with 0.2 U µl^−1^ RNAse inhibitors. Fc receptors were blocked, and fixed cells were labeled with the following antibodies for 30 min at 4 °C: mouse anti-NeuN AF488 (Merck Millipore, MAB377X; 1:100), rat anti-CD45 PE-Cy7 (BioLegend, 103114; 1:100), rat anti-CD11b-PE (BD Biosciences, 557397; 1:100) and mouse anti-O1-AF700 (R&D Systems, FAB1327N; 1:100). Single cells were identified by DAPI stain. Cells were washed twice, single DAPI^+^ cells were gated and around 100,000 cells were collected per sample using an SH800 sorter and corresponding software (Sony Biotechnology). Fixed cells from archived human brain tissue samples were counted and loaded without sorting.

### scRNA-seq and data processing

For scRNA-seq, we performed several independent experiments (three adult and three aged mice per reaction with two pooled reactions for live cells; three adult and three aged mice per reaction with two pooled reactions for fixed tissue; two adult, two aged and two PLX5622-treated aged mice per reaction with two multiplexed reactions for frozen fixed tissue; four adult and six aged human brain samples in two multiplexed reactions). Live single cells were sorted and counted (Countess 3 FL; Thermo Fisher Scientific) before being encapsulated into droplets with the Chromium Controller (10x Genomics) and processed according to the manufacturer’s specifications and previously published protocols^57^. Fixed single cells were sorted and counted or directly counted before probe hybridization with barcodes for multiplexing, pooling, washing and loading into the Chromium X (10x Genomics). Complementary DNA libraries ready for sequencing on Illumina platforms were generated using the Chromium Single-Cell 3′ Library & Gel Bead Kit v3.1 (10x Genomics) or the Chromium Fixed RNA Kit (10x Genomics) according to the detailed protocol provided by the manufacturer. Libraries were quantified by Qubit 3.0 Fluorometer (Thermo Fisher Scientific), and quality was checked using a 2100 Bioanalyzer with High Sensitivity DNA Kit (Agilent Technologies). Libraries were pooled and sequenced with a NovaSeq 6000 platform (S1 Cartridge; Illumina) in paired-end mode. The reads were aligned to the University of California, mouse mm10 or human hg38 reference genomes, and data were demultiplexed using CellRanger software (v.7.1.0). Subsequent data analysis was performed using the R package Seurat (v.5.0)^70^. Mouse datasets (processed and sequenced together) were merged, while human datasets were integrated with Harmony^71^ to correct for batch effects. Doublets and low-quality cells were removed based on the percentage of mitochondrial genes (cutoff set at 5%) and the number of genes (cells with >200 and <6,000 genes were used) expressed in each cell as quality control markers. The gene expression of the remaining cells was log-normalized. Highly variable genes were detected with Seurat, and the top 2,000 of these genes were used as the basis for downstream clustering analysis. Data were scaled, principal component analysis was used for dimensionality reduction and the number of principal components was identified using the built-in Elbow plot function. Cells were clustered based on the identified principal components; uniform manifold approximation and projection (UMAP) was used for data visualization in two dimensions. Cell clusters of interest were subset based on marker gene expression and reanalyzed. Differentially expressed genes were identified using the FindMarkers function. Complete lists of differentially expressed genes (*P*_adj_ < 0.05 after Bonferroni correction) are included in Supplementary Tables 1 and 2. Marker gene scores for feature expression programs were calculated using the AddModuleScore function in Seurat. Compositional analysis was done using scCODA (v.0.1.9)^72^ according to the online vignette (https://sccoda.readthedocs.io/en/latest/getting_started.html) with a false discovery rate set at 0.4. Gene-set enrichment analysis was performed using Metascape (v.3.5 (ref. ^73^); https://metascape.org). Cell–cell communication analysis of fixed scRNA-seq data was performed using CellChat^74^.

### MERFISH and data analysis

Optic nerves were cut as 10-µm-thick transverse or longitudinal sections and collected onto glass slides supplied by Vizgen. To ensure proper adhesion, the glass slides were coated with a solution of 0.1 mg ml^−1^ poly-d-lysine bromide (Sigma-Aldrich, P7886), left to incubate at room temperature for 1 h and dried before starting the sectioning. The gene panel used in this study included 496 protein-coding genes, complemented by 54 blank probes (Supplementary Table 3). Within this gene panel, a diverse array of transcripts comprising recognized markers for glial and immune cell types was included based on previously published literature. Sections were postfixed with 4% PFA in PBS for 15 min at room temperature, washed three times with PBS and then placed in the autofluorescence bleacher (Vizgen) for 3 h^52^. Afterward, the sections were permeabilized in 70% ethanol at 4 °C overnight. For hybridization with the gene panel, samples were washed with a sample prep wash buffer (Vizgen) and then incubated in a formamide hybridization buffer at 37 °C for 30 min before adding 50 μl of the gene panel mix on top of the tissue. Sections were hybridized at 37 °C for 36–48 h, washed and embedded into a polyacrylamide gel according to the manufacturer’s instructions. Samples were cleared with Clearing Premix containing proteinase K, washed and stained with DAPI and polyT reagent for 15 min at room temperature. The samples were then washed, and the appropriate hybridization and imaging buffers were loaded onto the MERSCOPE system (Vizgen). A low-resolution mosaic was acquired using a ×10 objective, and regions of interest were selected for high-resolution imaging with a ×60 lens. For high-resolution imaging, the focus was locked to the fiducial fluorescent beads on the coverslip. Seven 1.5-μm-thick *z* planes were imaged for each field of view. Raw images were decoded to RNA spots with spatial coordinates and gene IDs using Merlin software (Vizgen) on the MERSCOPE instrument. Cell segmentation was performed using the Cellpose^75^ algorithm based on the DAPI nuclear and polyT total RNA staining channels. The resulting single-cell gene expression matrices were further analyzed in the R package Seurat (v.5.0)^70^. Quality control for each section was performed, and we excluded cells with fewer than five detected genes, more than 750 detected transcripts and cell volume smaller than 100 µm^3^. Data were then normalized using SCTransform normalization; principal component analysis (PCA) was calculated based on all 496 measured genes, and UMAP embedding was computed from the first 30 PCA dimensions using the RunUMAP function. We calculated the shared nearest neighbor graph based on the first 30 PCA dimensions (FindNeighbors function), which was then used to detect clusters using a Louvain algorithm at a range of resolutions. We annotated cells into major classes based on marker genes and regional identity. We used the AddModuleScore function to calculate the glial activation scores as indicated in Fig. 6e. To identify differentially expressed genes in the T cell neighborhood, we calculated the 50 nearest neighbor cells of every T cell based on two-dimensional spatial coordinates of cell centroids with the BiocNeighbors package. We then identified differentially expressed genes between T cell neighbors and the remaining cells (non-neighbors) using the FindMarkers function with a Wilcoxon rank-sum test.

### Bone marrow transplantation

Bone marrow was transferred according to previously published protocols^22^. Briefly, bone marrow was isolated from the femur and tibia of donor mice, and 1 × 10^7^ cells were injected intravenously into anaesthetized *Rag1*^−/−^ mice; this provides a niche for engraftment and long-term reconstitution of adaptive immune cells and abolishes the need for confounding irradiation^22,76^. *Rag1*^−/−^ mice were reconstituted at 20 months of age and killed at 22 or 24 months of age. Successful bone marrow chimerism was controlled by flow cytometry of splenocytes and immunofluorescence in optic nerve sections.

### Spectral domain OCT

Mice were subjected to OCT imaging with a commercially available device (SPECTRALIS OCT; Heidelberg Engineering), a custom mount and additional lenses as described previously^77^. Mice were anesthetized by intraperitoneal injection of Ketavet and Xylavet, and their pupils were dilated by topical administration of one drop of 0.5% tropicamide eye drops (Mydrum; Bausch & Lomb) before image acquisition. Air-corneal interface refraction and corneal dehydration were prevented by applying artificial tears (Corneregel Fluid; Bausch & Lomb) and a custom-made polymethylmethacrylate hard contact lens (afocal, curvature—1.7 mm, diameter—3.2 mm; Cantor + Nissel). The thickness of the innermost retinal composite layer (combined nerve fiber layer (NFL), ganglion cell layer (GCL) and inner plexiform layer (IPL)) was measured in high‐resolution peripapillary circle scans (at least ten measurements per scan were averaged) by an investigator blinded for the genotype, age and treatment condition of the mice using HEYEX (v.1.7.1). The circle scans with automatic real-time tracking (100) were centered on the optic disc. Manual measurements were performed at 600× and 1:1 μm view in the proprietary software along the width of the circle scan, avoiding measurements on top of major blood vessels.

### Analysis of visual acuity

The visual acuity of mice was analyzed using automated optokinetic reflex tracking in an OptoDrum device (Striatech) as described previously^57^. Briefly, mice were placed on an elevated platform surrounded by monitors and a stripe pattern with maximum contrast and constant rotation speed (12° s^−1^) was presented. Optokinetic reflex behavior was automatically detected and analyzed by OptoDrum software (v.1.2.6) in an unbiased manner, and the stimulus pattern (cycles) was continuously adjusted to find the threshold of the animal’s visual acuity.

### Expansion microscopy

Super-resolution fluorescence microscopy was performed with 30-μm‐thick longitudinal optic nerve cryo-sections as described previously^57^. Free-floating sections were blocked using 5% BSA and 5% donkey serum in PBS containing 0.3% Triton X-100 and incubated overnight at 4 °C with a combination of the following antibodies: rat anti‐CD8 (Bio‐Rad Laboratories, MCA609G; 1:300), rabbit anti-GZMB (Abcam, ab4059; 1:100) and hamster anti-CD11c (Thermo Fisher Scientific, MA11C5; 1:100). Labeling was visualized using AF488 donkey anti-rat (Thermo Fisher Scientific, A-21208, 1:300), CF640R donkey antirabbit (Biotium, 20178; 1:300) and AF555 goat antihamster (Thermo Fisher Scientific, A78964, 1:300). Proteins were anchored using 0.1 mg ml^−1^ acryloyl-X (Thermo Fisher Scientific, A20770) in PBS for 24 h at room temperature. Sections were washed, partially air-dried on an uncharged slide, incubated in gelling solution (8.6 g per 100 ml sodium acrylate (Sigma-Aldrich, 408220), 2.5 g per 100 ml acrylamide (Sigma-Aldrich, A8887), 0.1 g per 100 ml N,N′-methylenebisacrylamide (Sigma-Aldrich, M7279), 11.7 g per 100 ml sodium chloride (Sigma-Aldrich, S6191), 0.2% TEMED accelerator solution (Sigma-Aldrich, T9281), 0.01% 4-hydroxy-TEMPO inhibitor solution (Sigma-Aldrich, 176141) and 0.2% ammonium persulfate (Sigma-Aldrich, 248614)) for 1 h at 4 °C. Afterward, sections were embedded in fresh gelling solution in an assembled chamber using coverslips as spacers and cover. Polymerization was performed at 37 °C for 2 h. Tissue-containing gels were trimmed, scooped off the slides and digested in proteinase K overnight at room temperature. Nuclei were labeled with DAPI, and gels were expanded by repeated washes in distilled water until expansion plateaued. Postexpansion sections were imaged using an LSM900 confocal microscope (ZEISS) with an AiryScan2 detector using an LD-C Apochromat ×40/1.1W objective. The expansion factor was calculated by measuring gels and labeled structures before and after expansion.

### Statistics and reproducibility

All quantifications and analyses were performed by blinded investigators who were unaware of the genotype, age and treatment group of the respective mice or tissue samples after concealment of groups with uniquely coded labels. Animals/samples were randomly placed into experimental or control groups according to the genotyping results using a random generator (http://www.randomizer.org). For biometrical sample size estimation, G*Power (v.3.1.3) was used^78^. Calculation of appropriate sample size groups was performed using a priori power analyses by comparing the mean of two to three groups with a defined adequate power of 0.8 (1 − *β* error) and an *α* error of 0.05. To determine the prespecified effect size *d* or *f*, previously published data were considered as comparable reference values^22^. The number of individual mice per group (number of biologically independent samples) for each experiment and the meaning of each data point are indicated in the respective figure legends. All data and micrographs represent at least three independent experiments with similar results. For the immunofluorescence analyses, we quantified a specific cell type/structure in at least three different sections of a respective tissue and averaged the measurements into one single data point. No animals or data were excluded from the analyses. Statistical analysis was performed using Prism 8 (GraphPad Software). The Shapiro–Wilk test was used to check for the normal distribution of data, and the *F* test was used to check the equality of variances to ensure that all data met the assumptions of the statistical tests used. Comparisons of two groups were performed with an unpaired Student’s *t* test (parametric comparison) or Mann–Whitney *U* test (nonparametric comparison). For multiple comparisons, a one‐way analysis of variance (ANOVA; parametric) or Kruskal–Wallis test (nonparametric) with Bonferroni’s post hoc test was applied, and adjusted *P* values are presented. *P* < 0.05 was considered statistically significant; exact *P* values are provided whenever possible in the figures (for post hoc tests) and figure legends (for null hypothesis testing) with three digits and a leading zero. Only *P* values smaller than 0.001 are shown as ‘*P* < 0.001’.

### Reporting summary

Further information on research design is available in the Nature Portfolio Reporting Summary linked to this article.

## Online content

Any methods, additional references, Nature Portfolio reporting summaries, source data, extended data, supplementary information, acknowledgements, peer review information; details of author contributions and competing interests; and statements of data and code availability are available at 10.1038/s41593-025-01955-w.

## Supplementary information

Supplementary Information Supplementary Figs. 1–10.

Reporting Summary

Supplementary Video Confocal microscopy allows the identification of individual CD11b^+^ DAPI^+^ microglia (1–5) in different Z-stack slices of a microglia cluster from an aged mouse optic nerve. Af, autofluorescent storage material. Scale bar, 10 µm.

Supplementary Data 1 Metascape gene-set enrichment analysis of upregulated WAM1 or WAM2 marker genes for scRNA-seq of live microglia (Extended Data Fig. 2). Top 100 enriched terms are shown.

Supplementary Data 2 Metascape gene-set enrichment analysis of upregulated WAM1, WAM2 or WAM3 marker genes for scRNA-seq of fixed microglia (Fig. 3). Top 100 enriched terms are shown.

Supplementary Data 3 Metascape gene-set enrichment analysis of upregulated genes comparing all oligodendrocytes from PLX5622-treated aged mice with aged mice for scRNA-seq of fixed oligodendrocytes (Extended Data Fig. 5). Top 100 enriched terms are shown.

Supplementary Table 1 Complete lists of cluster‐enriched markers and differentially expressed genes for scRNA‐seq data of live microglia (Extended Data Fig. 2).

Supplementary Table 2 Complete lists of cluster‐enriched markers and differentially expressed genes for scRNA‐seq data of fixed microglia (Fig. 3).

Supplementary Table 3 List of all targeted genes in the gene panel used for MERFISH (Fig. 6).

## Source data

Source Data Fig. 1 Statistical source data.

Source Data Fig. 2 Statistical source data.

Source Data Fig. 4 Statistical source data.

Source Data Fig. 5 Statistical source data.

Source Data Fig. 7 Statistical source data.

Source Data Fig. 8 Statistical source data.

Source Data Extended Data Fig. 1 Statistical source data.

Source Data Extended Data Fig. 4 Statistical source data.

Source Data Extended Data Fig. 5 Statistical source data.

Source Data Extended Data Fig. 8 Statistical source data.

Source Data Extended Data Fig. 9 Statistical source data.

Source Data Extended Data Fig. 10 Statistical source data.

## Data Availability

The sequencing data generated in this study have been deposited in the Gene Expression Omnibus (accession codes GSE283362 for fixed RNA profiling and GSE275954 for MERFISH). The indexed human reference genome (GRCh38) can be downloaded at the 10x Genomics website (https://cf.10xgenomics.com/supp/cell-exp/refdata-gex-GRCh38-2024-A.tar.gz). The indexed mouse reference genome (GRCm39) can be downloaded at the 10x Genomics website (https://cf.10xgenomics.com/supp/cell-exp/refdata-gex-GRCm39-2024-A.tar.gz). Source data are provided with this paper. Schemes for selected figure panels (Fig. 8a, Extended Data Fig. 8a, Supplementary Fig. 1a, Supplementary Fig. 3a, Supplementary Fig. 5a, Supplementary Fig. 7b) were created in BioRender.com. Source data are provided with this paper.

## Code availability

No previously unreported custom code for data analysis was created. All analyses were conducted following the established workflows for the R package Seurat^70^ as detailed on GitHub at https://github.com/Ruoqing-feng/Aging-Optic_nerve_MERFISH_scRNA-seq (ref. ^79^). Further details of the analysis or specific Seurat objects will be made available upon request.
