# Supplementary material for: Microglia activation orchestrates CXCL10-mediated CD8+ T cell recruitment to promote aging-related white matter degeneration
Source: Nat Neurosci. 2025 May 22;28(6):1160–73. doi: 10.1038/s41593-025-01955-w (PMC12148934; doi:10.1038/s41593-025-01955-w)
Supplement: Supplementary file 1 — Supplementary Figs. 1–10. [file 41593_2025_1955_MOESM1_ESM.pdf]

nature neuroscience

Supplementary information https://doi.org/10.1038/s41593-025-01955-w

Microglia activation orchestrates CXCL10-mediated CD8$^+$ T cell recruitment to promote aging-related white matter degeneration

In the format provided by the authors and unedited

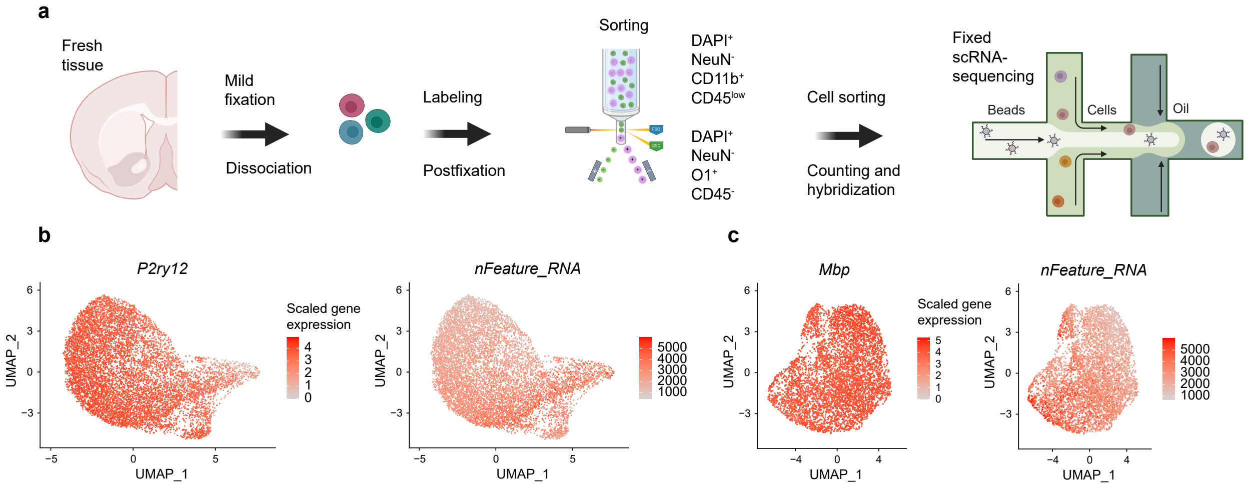

Supplementary Fig. 1 | Fixed scRNA-seq of microglia and oligodendrocytes in aging. a, Schematic experimental design. Created with BioRender. **b**, Feature plots of a microglia marker (*P2ry12*, left) and number of unique genes detected per cell (right) for fixed microglia sorted from adult (12-month-old) and aged (24-month-old) mouse brains as annotated in Extended Data Fig. 2f. **c**, Feature plots of an oligodendrocyte marker (*Mbp*, left) and number of unique genes detected per cell (right) for fixed oligodendrocytes sorted from adult and aged mouse brains as annotated in Supplementary Fig. 2a.

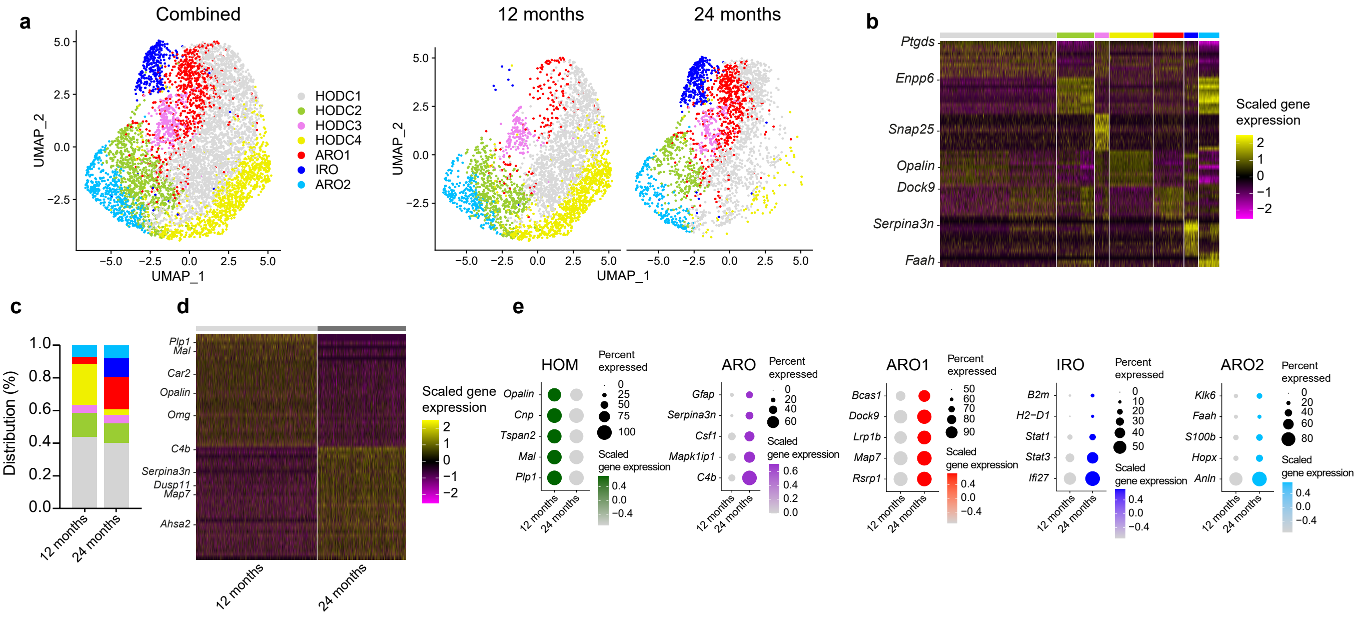

Supplementary Fig. 2 | scRNA-seq reveals aging-related oligodendrocyte signatures. a, UMAP visualization of fixed oligodendrocytes sorted from adult (12-month-old) and aged (24-month-old) mouse brains and analyzed by scRNA-seq. Combined (left, 6,104 cells) and separate (right) visualization of cells from adult (3,521 cells) and aged (2,583 cells) brains are displayed (n = 2 mice per group). **b**, Heatmap of top 10 cluster-specific genes. The color scale is based on a z-score distribution from -2 (purple) to 2 (yellow). **c**, Distribution of different cluster frequencies among all oligodendrocytes. **d**, Heatmap of top 30 differentially expressed genes comparing oligodendrocytes isolated from adult and aged brains across all clusters as identified in panel a. **e**, Dot plot expression visualization of selected genes implicated in oligodendrocyte homeostasis (HOM), aging response (ARO), and cell stress (STR), as well as selected marker genes of the distinct ARO and interferon-stimulated (IRO) states across all oligodendrocyte clusters. The color scales are based on z-score distributions. HODC, homeostatic oligodendrocytes; ARO, aging-related oligodendrocytes; IRO, interferon-responsive oligodendrocytes.

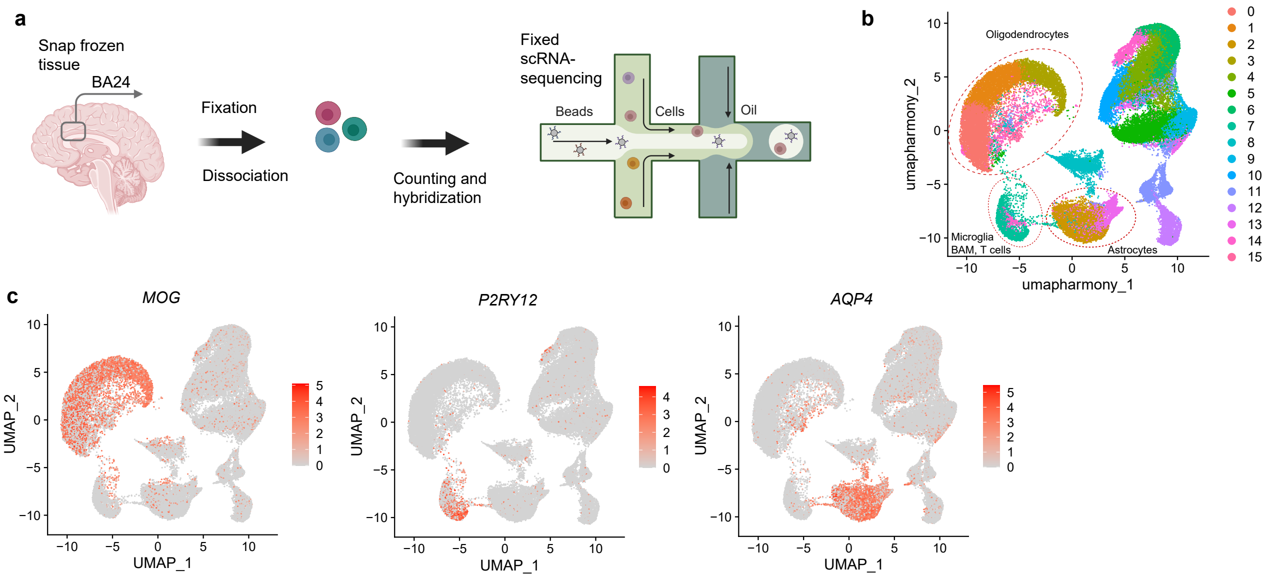

Supplementary Fig. 3 | Fixed scRNA-seq of glia and T cells in human aging. a Schematic experimental design. Created with BioRender. **b**, UMAP visualization of all fixed cells isolated from adult (25-50-year-old) and aged (>70-year-old) human brain samples and analyzed by scRNA-seq. **c**, Feature plots of oligodendrocyte (*MOG*, left), microglia (*P2RY12*, middle), and astrocyte (*AQP4*, right) markers for fixed subpopulations isolated from adult and aged human brain samples as annotated in panel b.

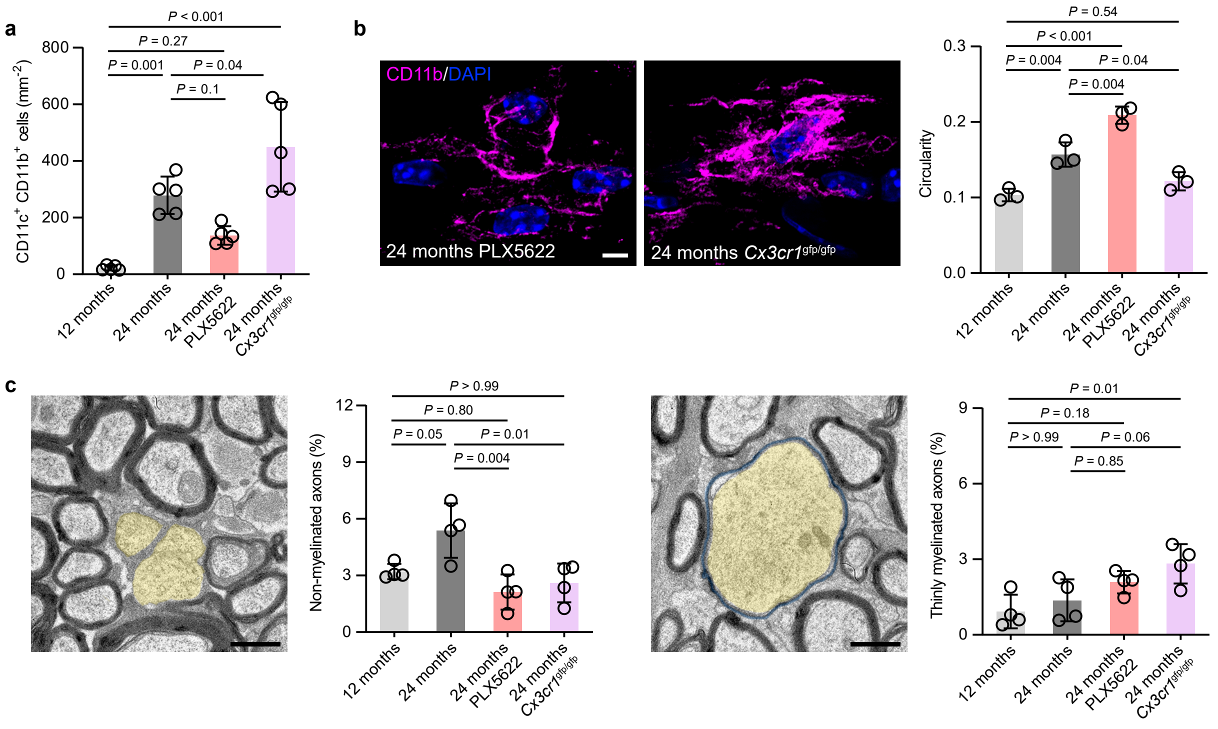

Supplementary Fig. 4 | Microglial response and myelin alterations upon PLX5622 treatment and CX3CR1 deficiency in aged optic nerves. **a**, Quantification of $CD11c^{+}$ $CD11b^{+}$ microglia in longitudinal optic nerve sections from adult (12-month-old), aged (24-month-old), PLX5622-treated aged, and *Cx3cr1*$^{gfp/gfp}$ aged mice (each circle represents the mean value of one mouse; $n = 5$ mice per group, one-way ANOVA with Bonferroni's multiple comparisons test, F (3, 16) = 22.27, $P < 0.001$). **b**, Immunofluorescence detection (left) and form factor analysis (right) of microglia in longitudinal optic nerve sections from adult, aged, aged PLX5622-treated, and aged Cx3cr1gfp/gfp mice ($n = 3$ mice per group, one-way ANOVA with Bonferroni's multiple comparisons test, F (3, 8) = 42.48, $P < 0.001$). **c**, Representative electron micrographs and quantifications of non-myelinated (left) and thinly myelinated (right) axons in optic nerve cross-sections from adult, aged, aged PLX5622-treated, and aged *Cx3cr1*$^{gfp/gfp}$ mice ($n = 4$ mice per group, one-way ANOVA with Bonferroni's multiple comparisons test, left: F (3, 12) = 7.946, $P = 0.003$ right: F (3, 12) = 5.680, $P = 0.01$); individual axons and a thin myelin sheath are pseudocolored. Scale bars, 2 µm. Data are presented as the mean ± s.d.

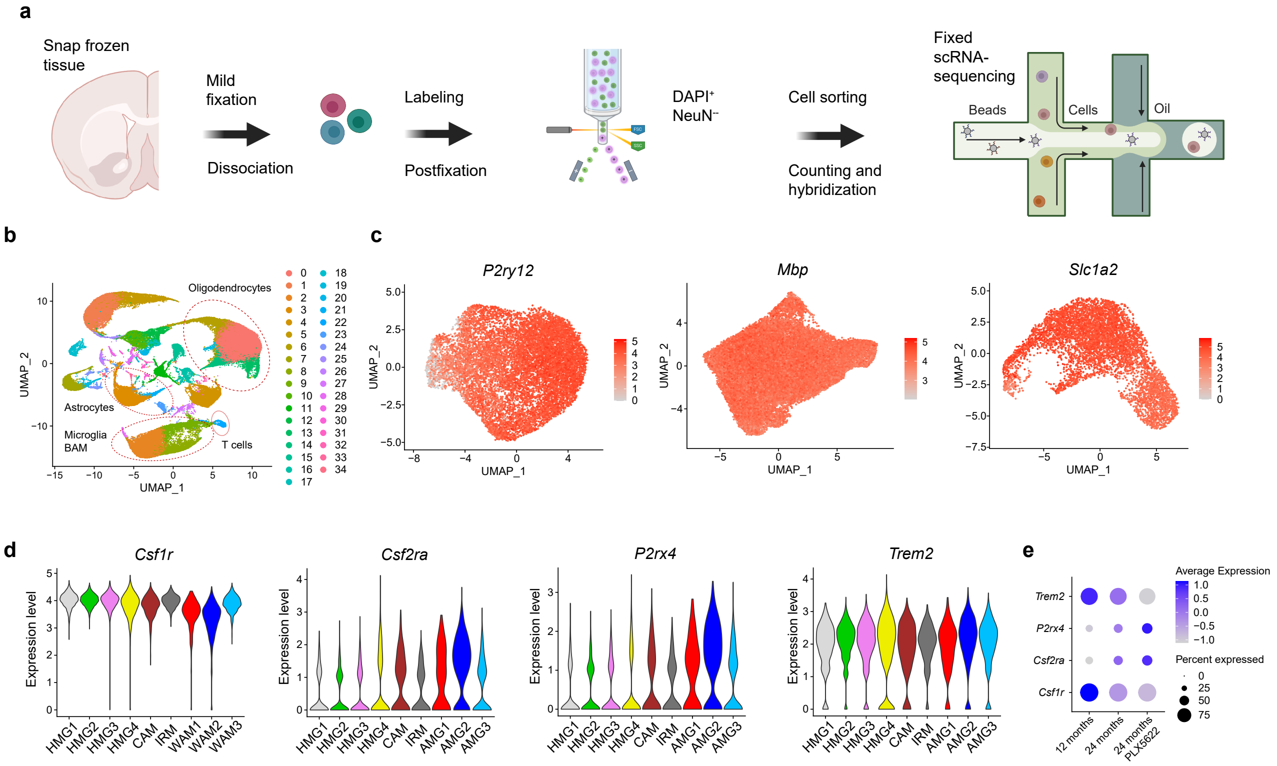

Supplementary Fig. 5 | Fixed scRNA-seq reveals aging-related glial signatures upon PLX5622 treatment. **a**, Schematic experimental design. Created with BioRender. **b**, UMAP visualization of all fixed cells (101,527 cells) sorted from adult (12-month-old), aged (24-month-old), and PLX5622-treated aged mouse brains and analyzed by scRNA-seq (n = 2 reactions per group with 2 mice per reaction). **c**, Feature plots of microglia (*P2ry12*, left), oligodendrocyte (*Mbp*, middle), and astrocyte (*Slc1a2*, right) markers for fixed subpopulations sorted from adult, aged, and PLX5622-treated aged mouse brains as annotated in panel b. **d**,Violin plots showing the expression of several genes encoding receptors implicated in microglial survival for subclusters as annotated in Fig. 3a. **e**, Dot plot showing expression of the same genes for all microglia in adult, aged, and PLX5622-treated aged mice.

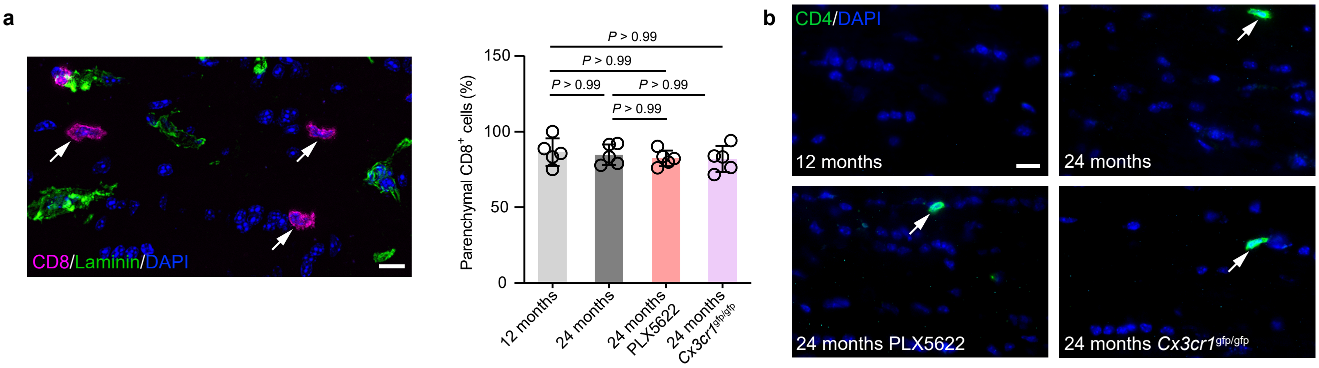

Supplementary Fig. 6 | T cell distribution upon PLX5622 treatment and CX3CR1 deficiency in aged optic nerves. a, Immunofluorescence detection (left) and quantification (right) of CD8$^+$ T cells associated with laminin$^+$ vessels or white matter parenchyma in longitudinal optic nerve sections from adult, aged, aged PLX5622-treated, and aged *Cx3cr1*$^{gfp/gfp}$ mice ($n = 5$ mice per group, one-way ANOVA with Bonferroni's multiple comparisons test, F (3, 16) = 0.4103, $P = 0.75$). Scale bar, 10 µm. **b**, Immunofluorescence detection of CD4$^+$ T cells (arrows) in longitudinal optic nerve sections from adult, aged, aged PLX5622-treated, and aged *Cx3cr1*$^{gfp/gfp}$ mice. Scale bar, 10 µm. Data are presented as the mean ± s.d.

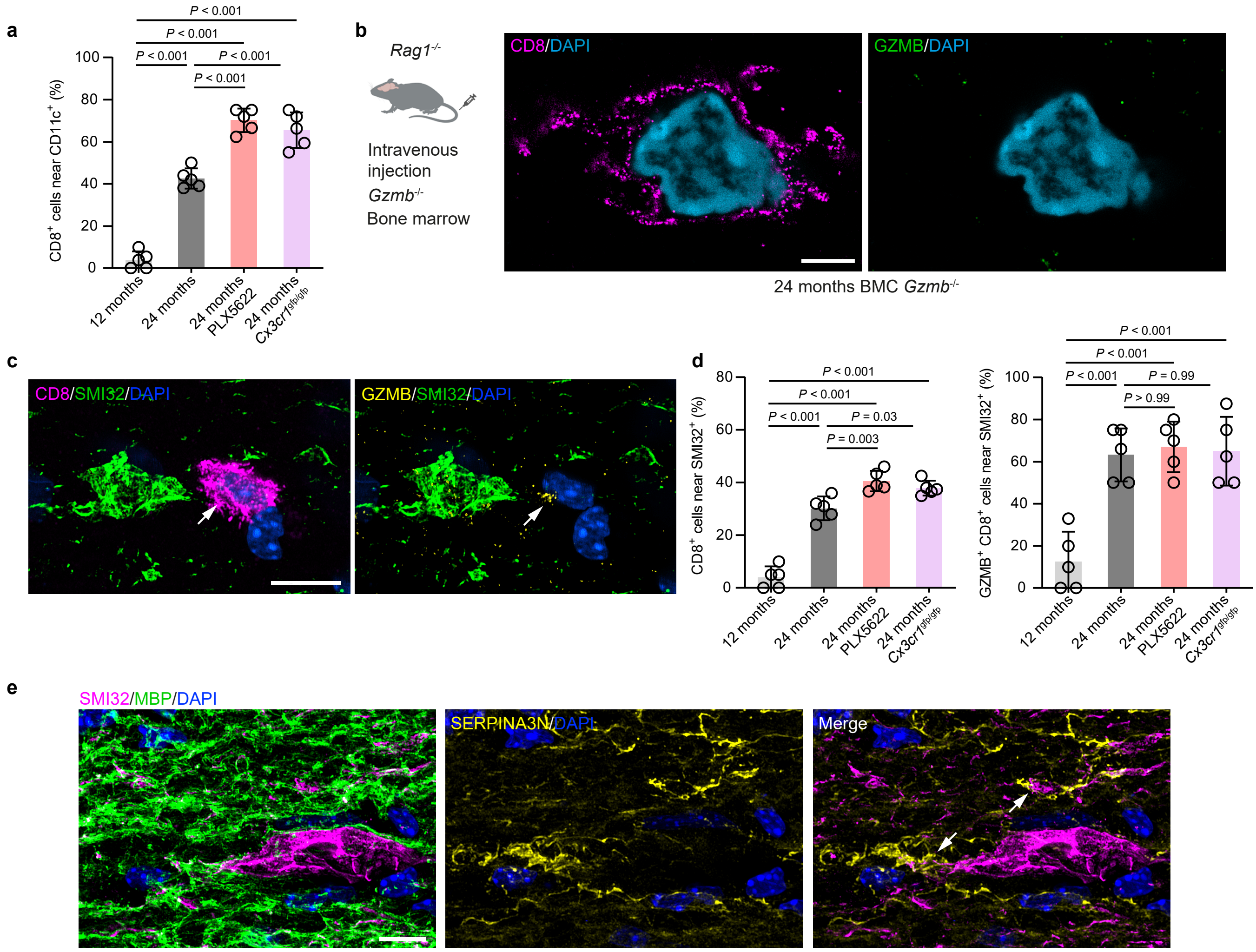

Supplementary Fig. 7 | Association of CD8+ T cells with reactive glia and damaged fibers in aged optic nerves. a, Quantification of the frequency of CD8+ T cells near CD11c+ microglia in longitudinal optic nerve sections from adult, aged, aged PLX5622-treated, and aged *Cx3cr1*$^{gfp/gfp}$ mice ($n = 5$ mice per group, one-way ANOVA with Bonferroni's multiple comparisons test, F (3, 16) = 130.0, $P < 0.001$). **b**, Super-resolution fluorescence detection of CD8 and GZMB in the optic nerve of an aged *Rag1*$^{-/-}$ mouse after transplantation with *Gzmb*$^{-/-}$ bone marrow demonstrates lack of GZMB+ granules in CD8+ T cells. Scale bar: 10 µm expanded, 2.5 µm unexpanded. **c**, Immunofluorescence detection and **d**, quantification of CD8+ T cells (left) and GZMB+ CD8+ T cells (arrow, right), in association with SMI32+ axonal spheroids in longitudinal optic nerve sections from adult, aged, aged PLX5622-treated, and aged *Cx3cr1*$^{gfp/gfp}$ mice ($n = 5$ mice per group, one-way ANOVA with Bonferroni's multiple comparisons test, left: F (3, 16) = 17.98, $P < 0.001$, right: F (3, 16) = 91.82, $P < 0.001$). Scale bar, 10 µm. **e**, Immunofluorescence detection of SMI32 and MBP in combination with SERPINA3N in longitudinal optic nerve sections from an aged mouse. Scale bar, 2 µm. Data are presented as the mean ± s.d.

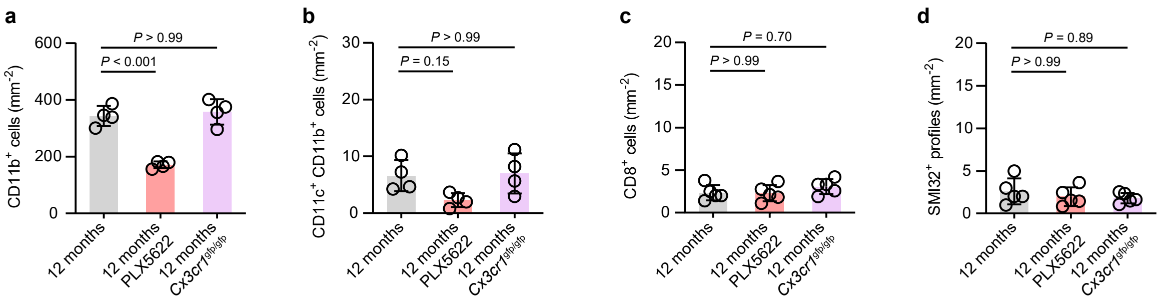

Supplementary Fig. 8 | Lack of microglial activation, T cell accumulation, and axonal damage upon PLX5622 treatment or CX3CR1 deficiency in adult mice. a, Quantification of CD11b$^+$ microglia, **b**, CD11c$^+$ microglia, **c**, CD8$^+$ T cells, and **d**, SMI32$^+$ axonal spheroids in longitudinal optic nerve sections from adult (12-month-old) PLX5622-treated, and *Cx3cr1*$^{gfp/gfp}$ mice (each circle represents the mean value of one mouse; n = 4-5 mice per group, one-way ANOVA with Bonferroni's multiple comparisons test, a: F (2, 9) = 38.10, P < 0.001; b: F (2, 9) = 3.776, P = 0.06, c: F (2, 12) = 1.109, P = 0.36; d: F (2, 12) = 0.6536, P = 0.54). Data are presented as the mean ± s.d.

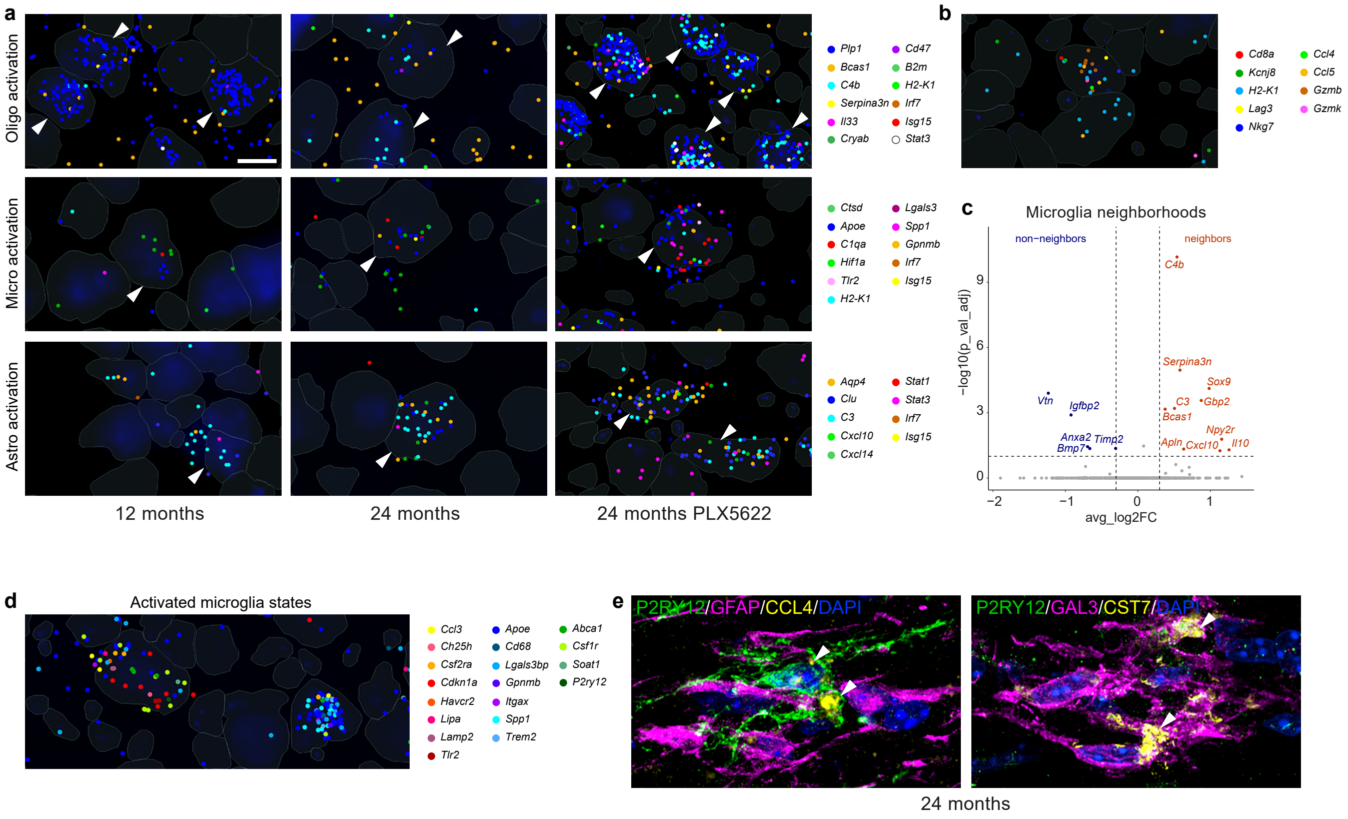

Supplementary Fig. 9: MERFISH- and immunofluorescence-based characterization of glial cells in aged white matter. a, Representative MERSCOPE visualizer images showing individual transcripts of selected genes representing cell type and activation markers for oligodendrocytes, microglia, and astrocytes in optic nerves from adult (12-month-old), aged (24-month-old), and PLX5622-treated aged mice. Cell segmentation boundaries are indicated and cells of interested are marked by arrowheads. Scale bar, 10 µm. **b**, Representative MERSCOPE visualizer image showing individual transcripts of selected genes representing cell type and effector markers for a $CD8^{+}$ T cells in the optic nerve from an aged (24-month-old) mouse. **c**, Volcano plot of differential gene expression between 50 nearest neighbors of microglia and non-neighboring cells. Bonferroni-adjusted P values from a Wilcoxon rank-sum test are shown. **d**, Representative MERSCOPE visualizer image showing individual transcripts of selected genes representing cell state and activation markers for distinct WAM populations in the optic nerve from an aged (24-month-old) mouse. **e**, Representative immunofluorescence detection of P2RY12 in combination with GFAP and CCL4 (left) or CST7 and GAL3 (right) in the optic nerve from an aged mouse demonstrate a $CCL4^{+}$ WAM1 (arrowheads) interacting with an astrocyte and a microglia nodule containing several WAM2 (arrowheads). Scale bar, 10 µm.

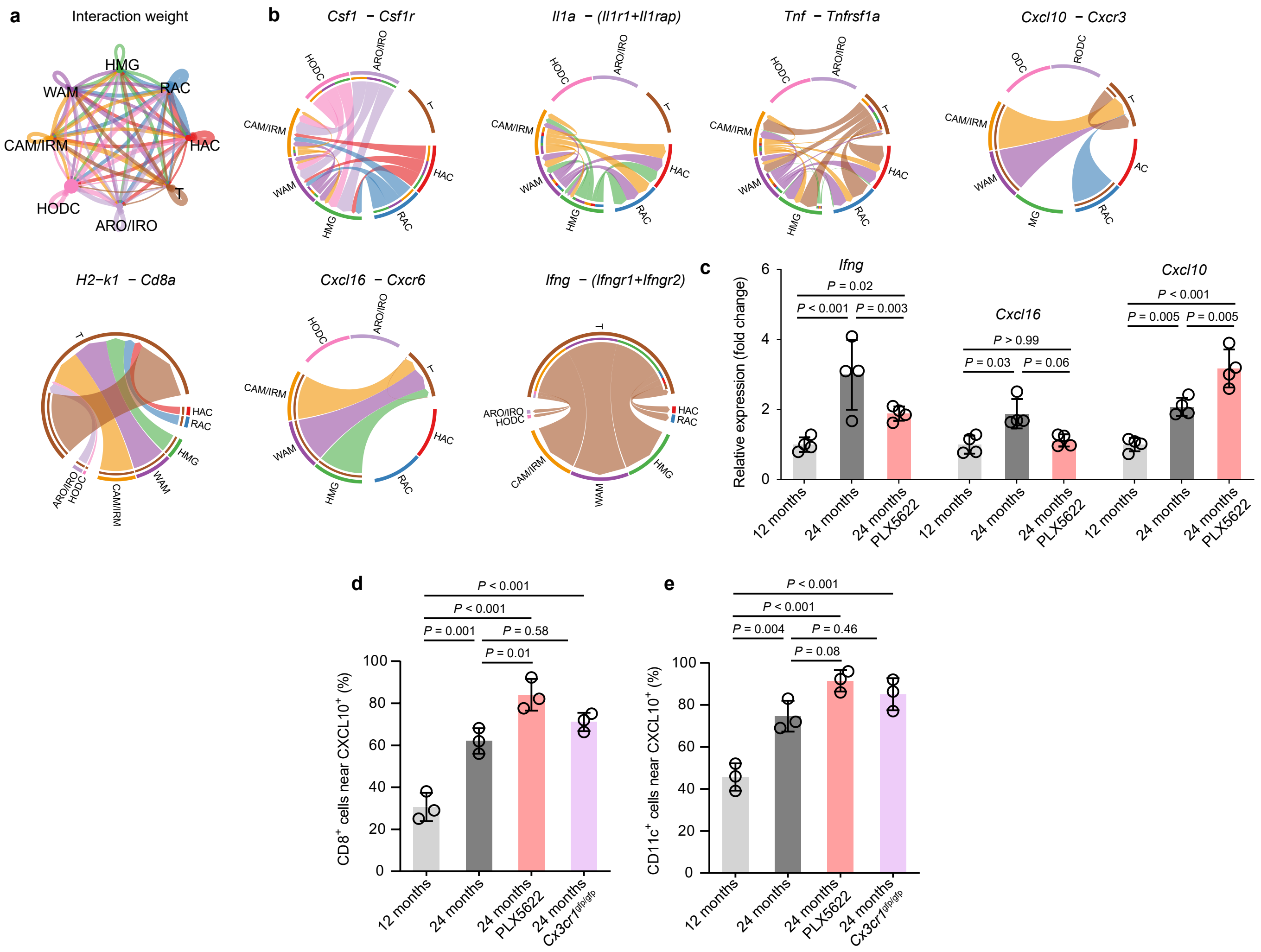

Supplementary Fig. 10 | Complex interactions between reactive glia and T cells in aging white matter. a, CellChat analysis showing the interaction weights of oligodendrocyte, microglia, astrocyte, and T cell clusters from fixed scRNA-seq. **b**, Chord diagrams showing selected ligand-receptor pairs possibly implicated in the interactions between reactive oligodendrocytes, microglia, astrocytes, and T cells. **c**, qRT-PCR of mRNA expression levels for selected transcripts in optic nerves from adult (12-month-old), aged (24-month-old), and PLX5622-treated aged mice (each circle represents the mean value of one mouse; $n = 4$ mice per group, two-way ANOVA with Bonferroni's multiple comparisons test, F (2, 27) = 30.10, $P < 0.001$). **d**, Immunofluorescence-based quantification of $CD8^+$ T cells or **e**, $CD11c^+$ microglia near $CXCL10^+$ cells in optic nerve longitudinal sections and *Cx3cr1*$^{gfp/gfp}$ mice (each circle represents the mean value of one mouse; $n = 3$ mice per group, one-way ANOVA with Bonferroni's multiple comparisons test, d: F (3, 8) = 39.38, $P < 0.001$; e: F (3, 8) = 27.4, $P < 0.001$). Data are presented as the mean ± s.d.
