# Supplementary material for: Microglia activation orchestrates CXCL10-mediated CD8+ T cell recruitment to promote aging-related white matter degeneration
Source: Nat Neurosci. 2025 May 22;28(6):1160–73. doi: 10.1038/s41593-025-01955-w (PMC12148934; doi:10.1038/s41593-025-01955-w)
Supplement: Supplementary file 2 — Reporting Summary [file 41593_2025_1955_MOESM2_ESM.pdf]

nature research

Corresponding author(s): Janos Groh

Last updated by author(s): Mar 10, 2025

Reporting Summary

Nature Research wishes to improve the reproducibility of the work that we publish. This form provides structure for consistency and transparency in reporting. For further information on Nature Research policies, see our Editorial Policies and the Editorial Policy Checklist.

Statistics

For all statistical analyses, confirm that the following items are present in the figure legend, table legend, main text, or Methods section.

n/a	Confirmed	
☐	☒	The exact sample size (*n*) for each experimental group/condition, given as a discrete number and unit of measurement
☐	☒	A statement on whether measurements were taken from distinct samples or whether the same sample was measured repeatedly
☐	☒	The statistical test(s) used AND whether they are one- or two-sided<br>*Only common tests should be described solely by name; describe more complex techniques in the Methods section.*
☐	☒	A description of all covariates tested
☐	☒	A description of any assumptions or corrections, such as tests of normality and adjustment for multiple comparisons
☐	☒	A full description of the statistical parameters including central tendency (e.g. means) or other basic estimates (e.g. regression coefficient) AND variation (e.g. standard deviation) or associated estimates of uncertainty (e.g. confidence intervals)
☐	☒	For null hypothesis testing, the test statistic (e.g. *F*, *t*, *r*) with confidence intervals, effect sizes, degrees of freedom and *P* value noted<br>*Give P values as exact values whenever suitable.*
☒	☐	For Bayesian analysis, information on the choice of priors and Markov chain Monte Carlo settings
☒	☐	For hierarchical and complex designs, identification of the appropriate level for tests and full reporting of outcomes
☐	☒	Estimates of effect sizes (e.g. Cohen's *d*, Pearson's *r*), indicating how they were calculated

Our web collection on statistics for biologists contains articles on many of the points above.

Software and code

Policy information about availability of computer code

Data collection	Striatech OptoDrum software (version 1.2.6), HeyEX (version 1.7.1), iTEM (version 5.1), Zen (version 2.3, blue edition), FacsDiva (version 6), CellRanger (version 7.1.0), Vizgen MERSCOPE software (version 233c)
Data analysis	Photoshop CS6 and Illustrator CS6 (Adobe Creative Suit 6), Fiji/ImageJ (version 1.51), FlowJo (version 10), IMARIS (v.9.7), STAR aligner (v.2.5.1b), R studio (version 2024.04.1), R (version 4.4.1), Seurat (version 5.0), Metascape (version 3.5), G*Power (version 3.1.3), Prism (version 8), CellChat version 1

For manuscripts utilizing custom algorithms or software that are central to the research but not yet described in published literature, software must be made available to editors and reviewers. We strongly encourage code deposition in a community repository (e.g. GitHub). See the Nature Research guidelines for submitting code & software for further information.

Data

Policy information about availability of data

All manuscripts must include a data availability statement. This statement should provide the following information, where applicable:

- Accession codes, unique identifiers, or web links for publicly available datasets
- A list of figures that have associated raw data
- A description of any restrictions on data availability

The sequencing data generated in this study have been deposited in the Gene Expression Omnibus (accession codes GSE283362 for fixed RNA profiling and GSE275954 for MERFISH). The indexed Human reference genome (GRCh38) can be downloaded at the 10x Genomics website (https://cf.10xgenomics.com/supp/cell-exp/refdata-gex-GRCh38-2024-A.tar.gz). The indexed Mouse reference genome (GRCm39) can be downloaded at the 10x Genomics website (https://cf.10xgenomics.com/supp/cell-exp/refdata-gex-GRCm39-2024-A.tar.gz). Source data are provided with this paper. Schemes for selected figure panels were created in BioRender. Groh, J. (2025) https://BioRender.com/y27r313.

Field-specific reporting

Please select the one below that is the best fit for your research. If you are not sure, read the appropriate sections before making your selection.

☒ Life sciences ☐ Behavioural & social sciences ☐ Ecological, evolutionary & environmental sciences

For a reference copy of the document with all sections, see nature.com/documents/nr-reporting-summary-flat.pdf

Life sciences study design

All studies must disclose on these points even when the disclosure is negative.

Sample size	For biometrical sample size estimation, the program G*Power (version 3.1.3) was used. Calculation of appropriate sample size groups was performed in a priori power analysis by comparing the mean of 2 to 4 groups with a defined adequate power of 0.8 (1 - beta-error) and an α-error of 0.05. To determine the prespecified effect size d or f, previously published data were considered as comparable reference values. For single cell RNA-seq and MERFISH, no sample size calculation was performed. The total number of single cells, batches, and sections analyzed was set to ensure that multiple biological and technical replicates contributed to the reported cell populations according to previous publications.
Data exclusions	All exclusion criteria during exploratory data analysis are described in the relevant section of the Methods and were not pre-established. No animals were excluded from the analyses. For scRNA-seq, data from low quality cells like doublets and potentially dead cells were removed based on the percentage of mitochondrial genes (cut-off set at 5%) and the number of genes (cells with >200 and <6000 genes were used) expressed in each cell as quality control markers. For flow cytometry, non-viable cells and doublets were excluded. These exclusions were necessary to ensure precision and reliability of the data.
Replication	Experiments were repeated with similar results at least two times with independent biological replicates. All replications were successful. For single cell RNA-seq and MERFISH, the total number of single cells, batches, and sections analyzed was set to ensure that multiple animals or human samples contributed to the reported cell populations and to allow the comparison of cells from all analyzed groups in the respective experiment.
Randomization	Animals were randomly placed into experimental or control groups according to genotyping results using a random generator (http://www.randomizer.org).
Blinding	All quantifications and behavioral analyses were performed by blinded investigators who were unaware of the genotype, age, and treatment group of the respective mice or tissue samples after concealment of this information with individual uniquely coded labels.

Reporting for specific materials, systems and methods

We require information from authors about some types of materials, experimental systems and methods used in many studies. Here, indicate whether each material, system or method listed is relevant to your study. If you are not sure if a list item applies to your research, read the appropriate section before selecting a response.

Materials & experimental systems

n/a	Involved in the study	
☐	☒	Antibodies
☒	☐	Eukaryotic cell lines
☒	☐	Palaeontology and archaeology
☐	☒	Animals and other organisms
☐	☒	Human research participants
☒	☐	Clinical data
☒	☐	Dual use research of concern

Methods

n/a	Involved in the study	
☒	☐	ChIP-seq
☐	☒	Flow cytometry
☒	☐	MRI-based neuroimaging

Antibodies

Antibodies used	Histochemistry and immunofluorescence<br>an appropriate combination of up to 3 of the following antibodies: rat anti-CD11b (1:100, catalog no. MCA74G; Bio-Rad Laboratories); hamster anti-CD11c (1:100, catalog no. MA11C5; Thermo Fisher Scientific); mouse anti-neurofilament H non-phosphorylated, SMI32 (1:1,000, catalog no. 801701; BioLegend); rat anti-CD8 (1:500, catalog no. MCA609G; Bio-Rad Laboratories); goat anti-GZMB (1:100, catalog no. AF1865; R&D Systems); rabbit anti-GZMB (1:100, catalog no. ab4059; abcam); rabbit anti-Laminin (1:300 catalog no. ab11575; abcam); rabbit anti-P2RY12 (1:300, catalog no. 55043A; AnaSpec); rabbit anti-GAL3 (1:1,000, NBP3-03252; Novus Biologicals); rat anti-GAL3 (1:300, catalog no. 125402; BioLegend); goat anti-CXCL10 (1:500, catalog no. AF-466-NA; R&D Systems); mouse anti-GFAP (1:1000, catalog no. G3893; Sigma-Aldrich); rabbit anti-TCF-1 (1:100, catalog no. 2203; Cell Signaling), goat anti-SERPINA3N (1:100, catalog no. AF4709; Bio-Techne; Retinae were incubated overnight on a rocker at 4 °C with guinea pig anti-RBPMS (1:300, catalog no. ABN1376; Merck Millipore) antibodies; Immunoreactive profiles were visualized using fluorescently labeled (1:300; Dianova or Thermo Fisher Scientific) secondary antibodies; nuclei were stained with 4,6-diamidino-2-

phenylindole (DAPI) (Sigma-Aldrich). rat anti-CD8 (1:300, catalog no. MCA609G; Bio-Rad Laboratories); rabbit anti-GZMB (1:100, catalog no. ab4059; abcam); hamster anti-CD11c (1:100, catalog no. MA11C5; Thermo Fisher Scientific); Labeling was visualized using AF488 donkey anti-rat (1:300, catalog no. A-21208; Thermo Fisher Scientific), CF640R donkey anti-rabbit (1:300, catalog no. 20178; Biotium), and AF555 goat anti-hamster (1:300, catalog no. A78964; Thermo Fisher Scientific)
Flow cytometry and cell sorting
rat anti-CD16/32 (1:200, catalog no. 553141; BD Biosciences); rat anti-CD45 PerCP/Cyanine5.5 (1:100, catalog no. 130-102-469; Miltenyi Biotec); rat anti-SiglecH PE (1:100, catalog no. 12-0333-82; eBioscience); rat anti-O1 AF700 (1:100, FAB1327N-100UG; R&D Systems); rat anti-CD11c APC (1:100, catalog no. 117310: BioLegend); rat anti-PD1 BV605 (1:100, catalog no. 135219; BioLegend); rat anti-CD11b-PE (1:100, catalog no. 557397; BD Biosciences); mouse anti-NeuN AF488 (1:100, catalog no. MAB377X; Merck Millipore); rat anti-CD45 PE-Cy7 (1:100, catalog no. 103114; BioLegend).

Validation

All primary antibodies were previously validated for the species and application (see citations provided on manufacturers websites for the above catalogue numbers) and/or in previous studies by the authors. Appropriate positive and negative controls or, if available, tissues from respective knockout mice were used. Immunoreactive profiles showed the typical morphology of the respective target cells/structures.

Animals and other organisms

Policy information about studies involving animals; ARRIVE guidelines recommended for reporting animal research

Laboratory animals

Mice were kept at the animal facility of the Centre for Experimental Molecular Medicine, University of Würzburg, or the German Center for Neurodegenerative Diseases (DZNE) in Munich under barrier conditions and at a constant cycle of 12 h in the light (<300 lux) and 12 h in the dark. Colonies were maintained at 20-24 °C and 40-60% humidity, with free access to food and water. All mice including wild type (WT), Cx3cr1gfp/gfp (B6.129P2(Cg)-Cx3cr1tm1Litt/J), Cd8-/- (B6.129S2-Cd8atm1Mak/J), Trem2-/- (B6.129P2-Trem2tm1cln/J), Cxcl10-/- (B6.129S4-Cxcl10tm1Adl/J), Rag1-/- (B6.129S7-Rag1tm1Mom/J), Cxcr3-/- (B6.129P2-Cxcr3tm1Dgen/J) were on a uniform C57BL/6J genetic background; they were bred, regularly backcrossed and aged in-house. Since we did not detect obvious differences between male and female mice in the analyses presented in the current study, mice of either sex were used for most experiments. For PLX5622 treatment and scRNA-seq experiments, male mice were used. Genotypes were determined by conventional PCR using isolated DNA from ear punch biopsies. Mice were analyzed at various ages as indicated in the respective experiments, including 2 months, 6 months, 12 months, 18, months, and 24 months of age.

Wild animals

This study did not involve wild animals.

Field-collected samples

This study did not involve samples collected from the field.

Ethics oversight

All animal experiments were approved by the Governments of Lower Franconia, Würzburg, Germany and of Upper Bavaria, Munich, Germany (AZ 55.2 DMS 2532-2-1; AZ 55.2 DMS 2532-2-399; AZ 55.2 DMS 2532-2-907; AZ 55.2 DMS 2532-2-1029; AZ 55.2 DMS 2532-2-1191). The procedures were performed according to international guidelines on the use of laboratory mice.

Note that full information on the approval of the study protocol must also be provided in the manuscript.

Human research participants

Policy information about studies involving human research participants

Population characteristics

Human brain autopsy samples from normal adults (25 to 50 years old) and elderly (70+ years old) of either sex without neurological disease were acquired from the Edinburgh Brain and Tissue Bank. Frozen samples of the anterior cingulate cortex (BA24) cortex were analyzed.

Recruitment

n/a. Human brain autopsy samples were acquired from the Edinburgh Brain and Tissue Bank.

Ethics oversight

The ethical committee of the University of Würzburg had no objections regarding the anonymous analysis of the material for scientific purposes.

Note that full information on the approval of the study protocol must also be provided in the manuscript.

Flow Cytometry

Plots

Confirm that:

☒ The axis labels state the marker and fluorochrome used (e.g. CD4-FITC).

☒ The axis scales are clearly visible. Include numbers along axes only for bottom left plot of group (a 'group' is an analysis of identical markers).

☒ All plots are contour plots with outliers or pseudocolor plots.

☒ A numerical value for number of cells or percentage (with statistics) is provided.

Methodology

Sample preparation

Mice were euthanized with CO_2 (according to the guidelines by the State Office of Health and Social Affairs Berlin) and blood was thoroughly removed by transcardial perfusion with PBS containing heparin. Brains including optic nerves, leptomeninges

and choroid plexus were dissected, collected in ice-cold PBS and cut into small pieces. Tissue was digested in 1 ml of Accutase (Merck Millipore) per brain at room temperature for 15 min and triturated through 70-µm cell strainers, which were rinsed with 10% FCS in PBS. Cells were purified by a linear 40% Percoll (GE Healthcare) centrifugation step at 650 g without brakes for 25 min and the myelin top layer and supernatant were discarded. Mononuclear cells were resuspended in 1% BSA in PBS and isolated cells were counted for each brain. Viable cells were identified by Calcein blue AM stain (catalog no. ABD-22007; Biomol), Fc receptors were blocked for 15 min with rat anti-CD16/32 (1:200, catalog no. 553141; BD Biosciences) and cells were washed and labeled with the described antibodies for 30 min at 4 °C.

Instrument	FACSAria III (BD Biosciences), FACSLyric (BD Biosciences), Sony SH800
Software	FacsDiva, Sony SH800 software, FlowJo (version 10)
Cell population abundance	Cell populations were FACS sorted in semi-purity mode. Purity was determined by analyzing sorted cells for the expression of marker genes for other cell types or poor quality by scRNA-seq.
Gating strategy	Gates were set manually by using compensation beads and appropriate control samples. Example gating strategies are provided in the corresponding Figures.

☒ Tick this box to confirm that a figure exemplifying the gating strategy is provided in the Supplementary Information.
