# Supplementary figures and images for: Microglia activation orchestrates CXCL10-mediated CD8+ T cell recruitment to promote aging-related white matter degeneration
Source: Nat Neurosci. 2025 May 22;28(6):1160–73. doi: 10.1038/s41593-025-01955-w (PMC12148934; doi:10.1038/s41593-025-01955-w)

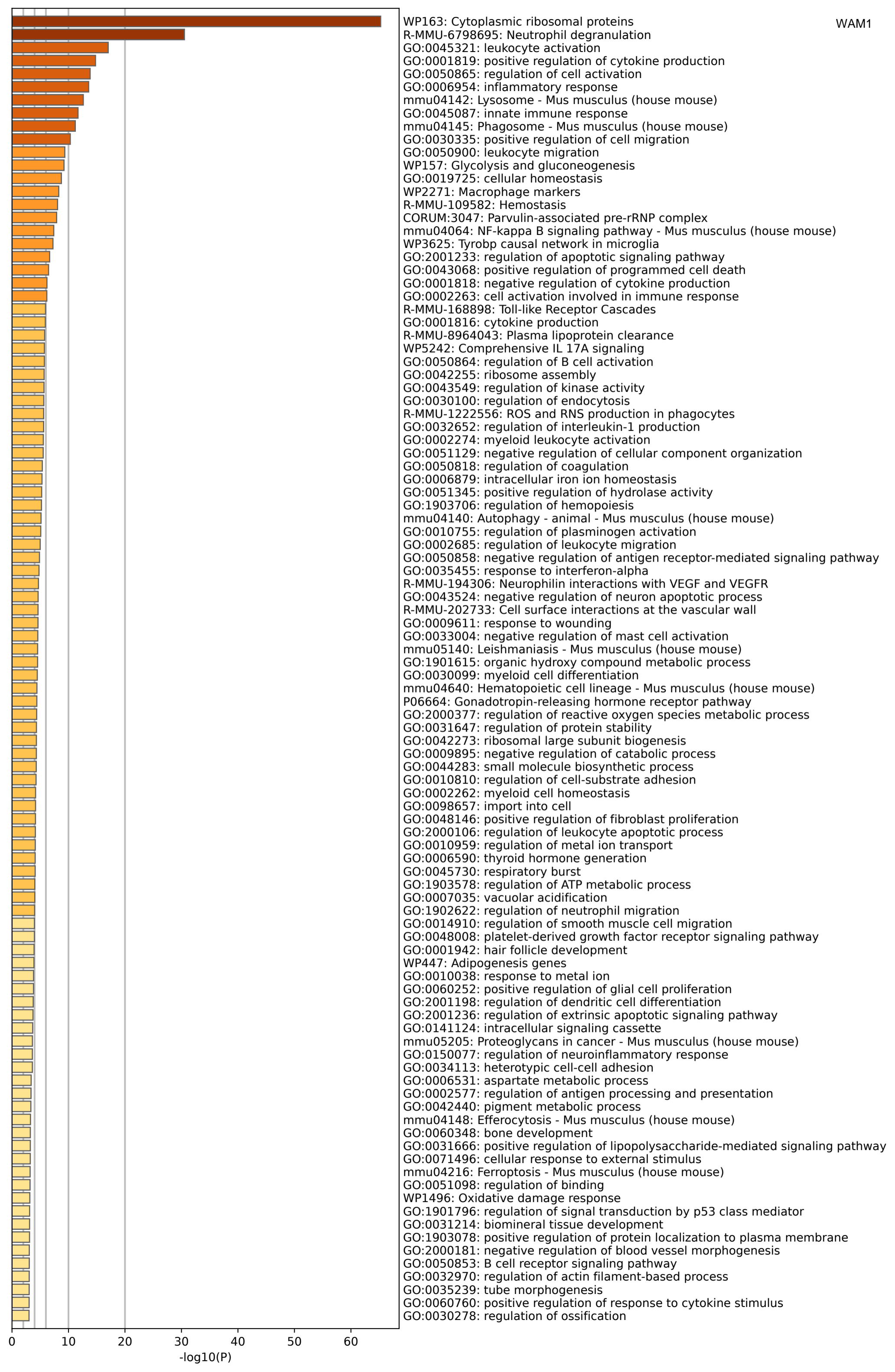

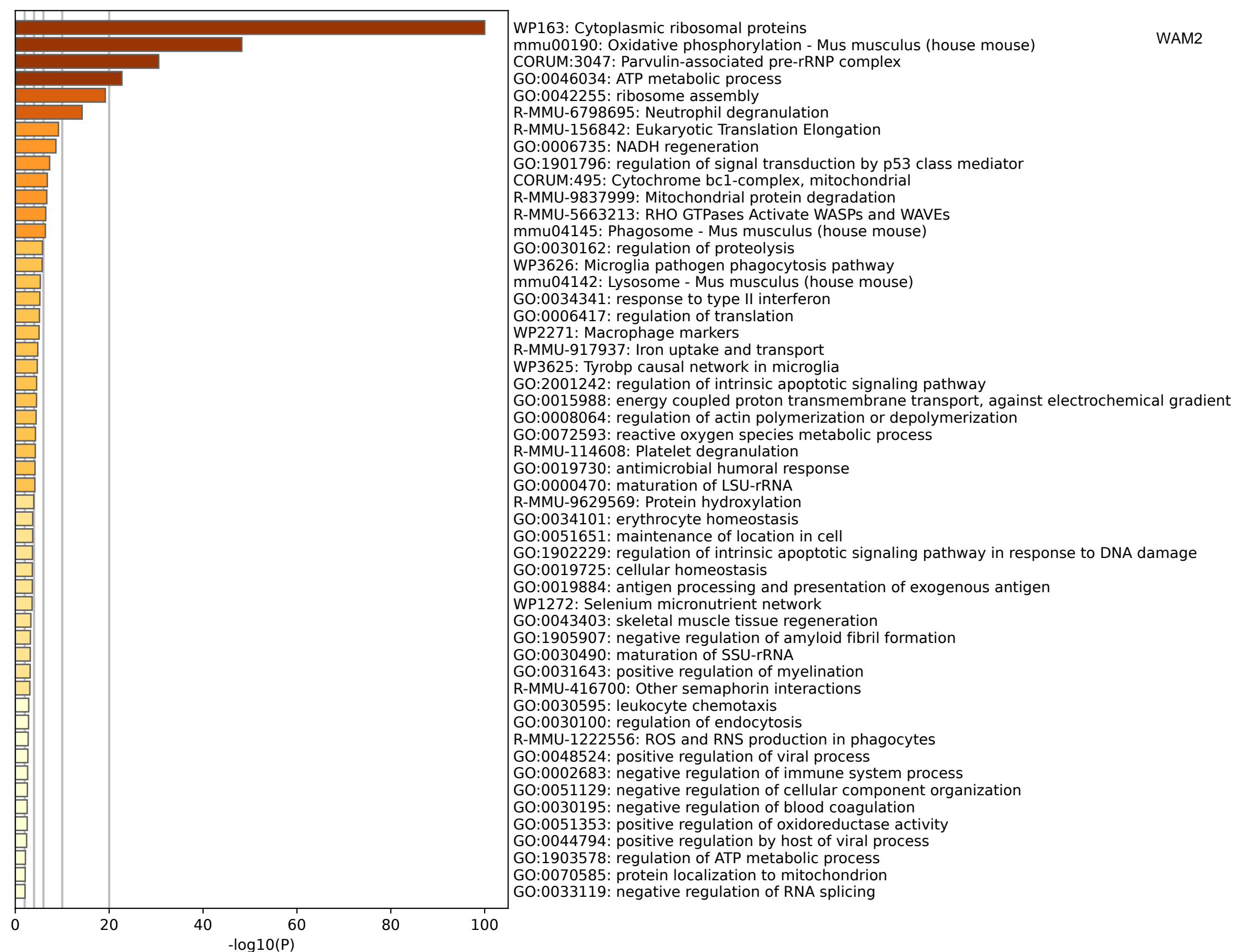

Supplement: Supplementary file 4 — Metascape gene-set enrichment analysis of upregulated WAM1 or WAM2 marker genes for scRNA-seq of live microglia (Extended Data Fig. 2). Top 100 enriched terms are shown. [file 41593_2025_1955_MOESM4_ESM.pdf]

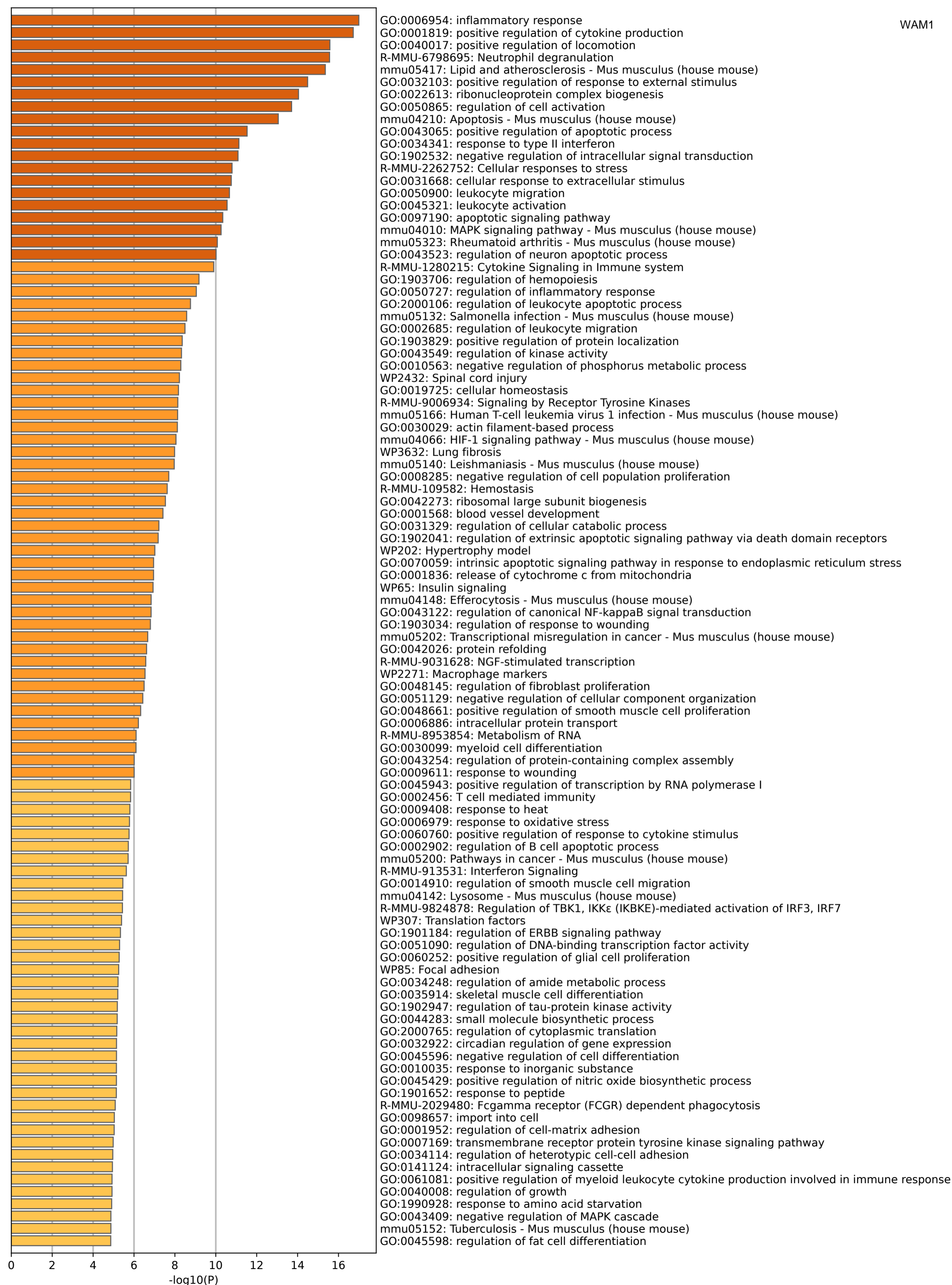

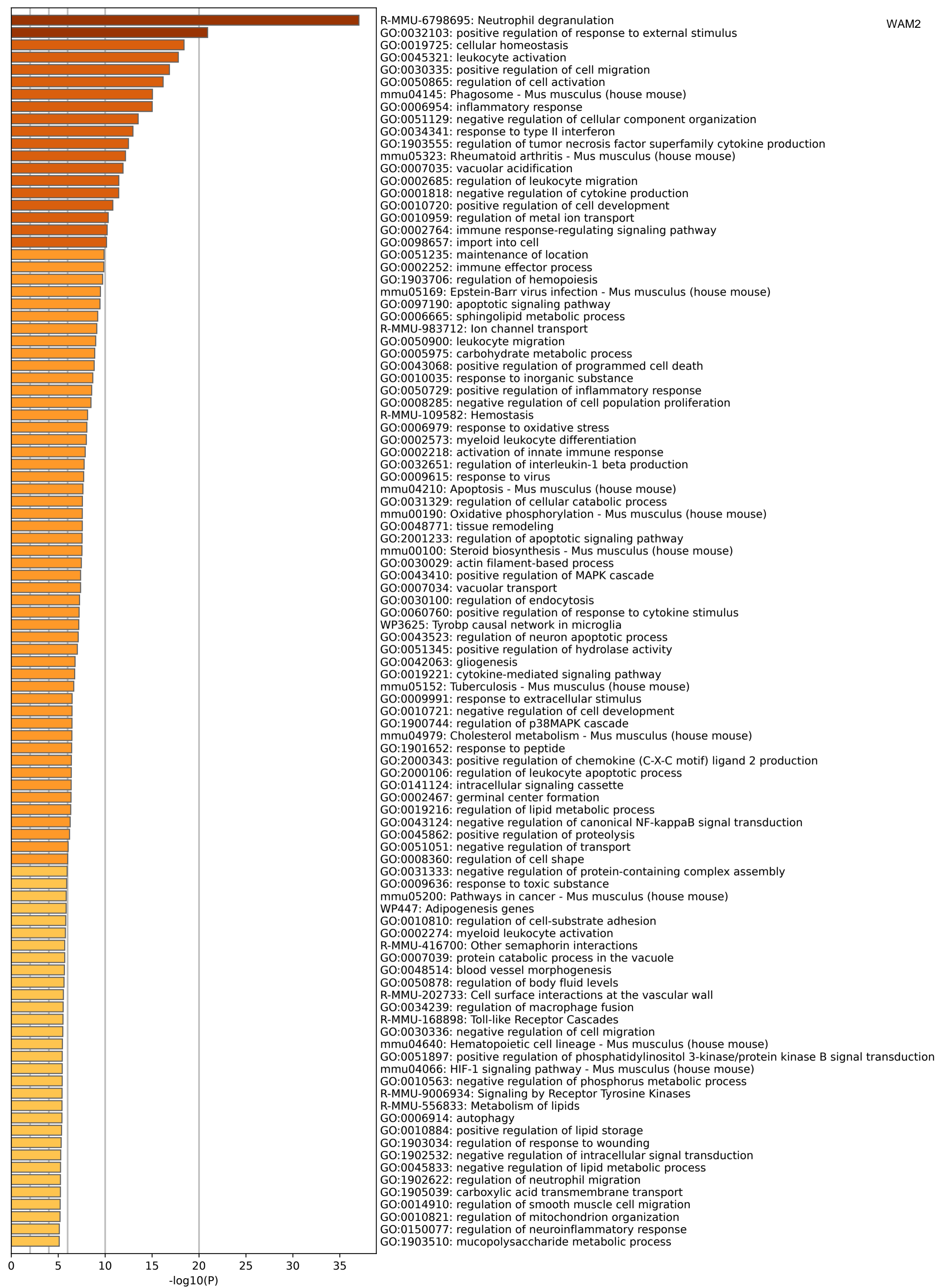

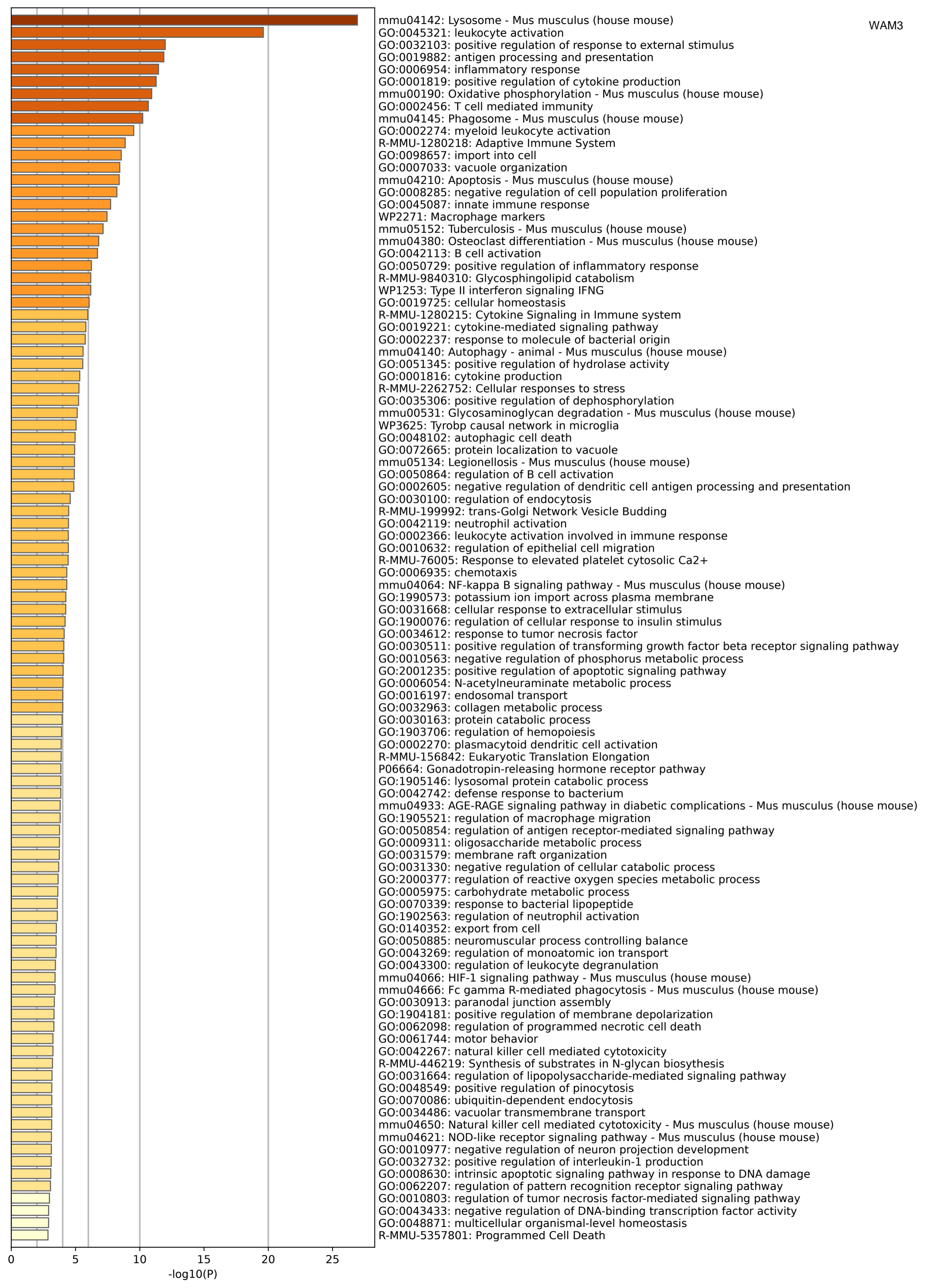

Supplement: Supplementary file 5 — Metascape gene-set enrichment analysis of upregulated WAM1, WAM2 or WAM3 marker genes for scRNA-seq of fixed microglia (Fig. 3). Top 100 enriched terms are shown. [file 41593_2025_1955_MOESM5_ESM.pdf]

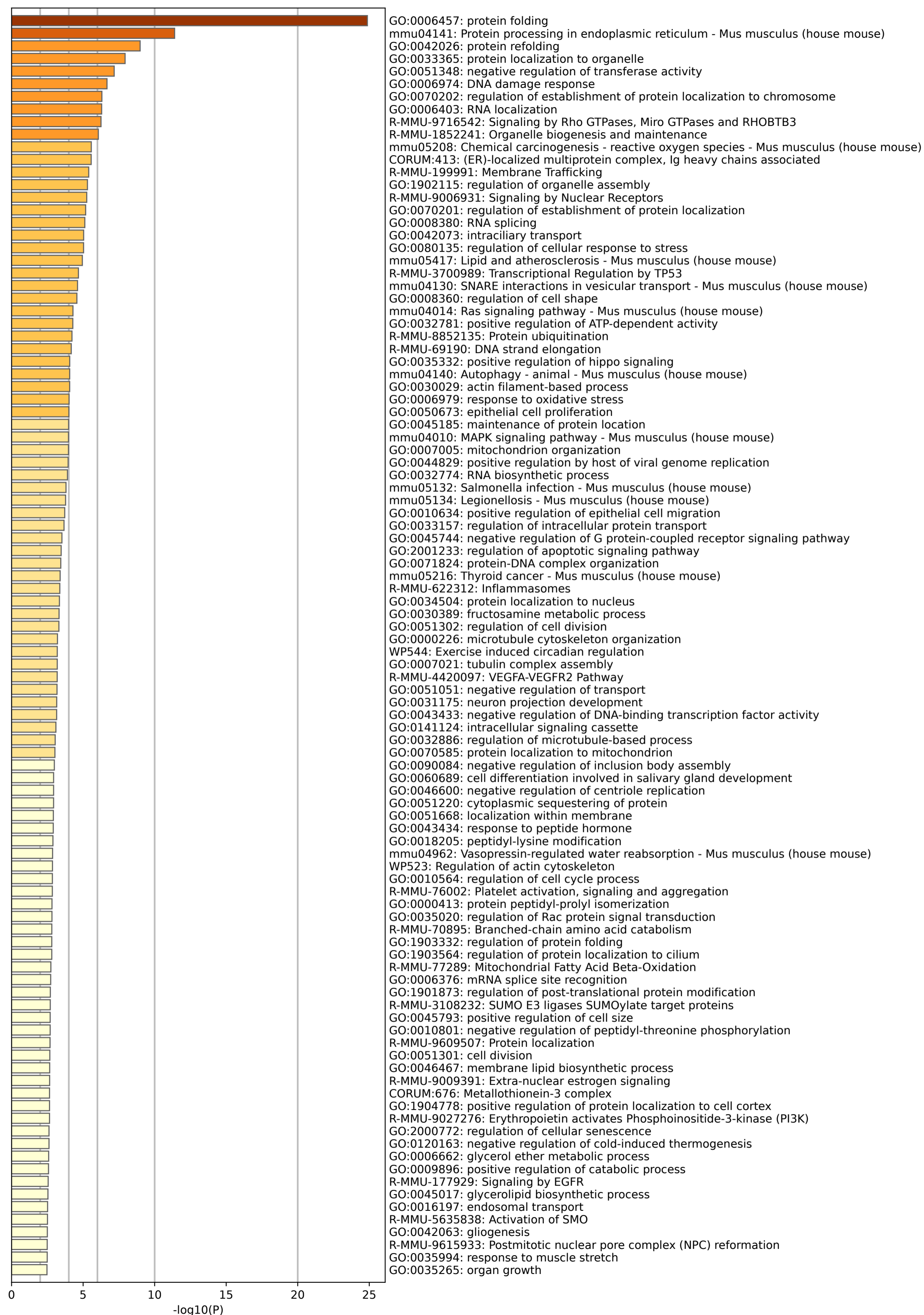

Supplement: Supplementary file 6 — Metascape gene-set enrichment analysis of upregulated genes comparing all oligodendrocytes from PLX5622-treated aged mice with aged mice for scRNA-seq of fixed oligodendrocytes (Extended Data Fig. 5). Top 100 enriched terms are shown. [file 41593_2025_1955_MOESM6_ESM.pdf]
